# Fully-automated segmentation of muscle and inter-/intra-muscular fat from magnetic resonance images of calves and thighs: an open-source workflow in Python

**DOI:** 10.1186/s13395-024-00365-z

**Published:** 2024-12-27

**Authors:** Kenneth Tam, Si Wen Liu, Sarah Costa, Eva Szabo, Shannon Reitsma, Hana Gillick, Jonathan D. Adachi, Andy Kin On Wong

**Affiliations:** 1 Department of Neurobiology, Physiology, and Behavior, University of California Davis, Davis, CA USA; 2 Rehabilitation Sciences Institute, University of Toronto, Toronto, ON Canada; 3 Joint Department of Medical Imaging, University Health Network, Toronto, ON Canada; 4 Department of Medicine, McMaster University, Hamilton, ON Canada; 5 Schroeder’s Arthritis Institute, University Health Network, Toronto, ON Canada; 6 Dalla Lana School of Public Health, University of Toronto, Toronto, ON Canada; 7 Toronto General Hospital Research Institute, 200 Elizabeth St. 7EN-238, Toronto, ON M5G2C4 Canada

**Keywords:** Muscle adiposity, Intramuscular fat, Intermuscular fat, MRI, Segmentation, Automated, Open source, Workflow

## Abstract

**Background:**

INTER- and INTRAmuscular fat (IMF) is elevated in high metabolic states and can promote inflammation. While magnetic resonance imaging (MRI) excels in depicting IMF, the lack of reproducible tools prevents the ability to measure change and track intervention success.

**Methods:**

We detail an open-source fully-automated iterative threshold-seeking algorithm (ITSA) for segmenting IMF from T1-weighted MRI of the calf and thigh within three cohorts (CaMos Hamilton (*N* = 54), AMBERS (*N* = 280), OAI (*N* = 105)) selecting adults 45–85 years of age. Within the CaMos Hamilton cohort, same-day and 1-year repeated images (*N* = 38) were used to evaluate short- and long-term precision error with root mean square coefficients of variation; and to validate against semi-automated segmentation methods using linear regression. The effect of algorithmic improvements to fat ascertainment using 3D connectivity and partial volume correction rules on analytical precision was investigated. Robustness and versatility of the algorithm was demonstrated by application to different MR sequences/magnetic strength and to calf versus thigh scans.

**Results:**

Among 439 adults (319 female(89%), age: 71.6 ± 7.6 yrs, BMI: 28.06 ± 4.87 kg/m^2^, IMF%: 10.91 ± 4.57%), fully-automated ITSA performed well across MR sequences and anatomies from three cohorts. Applying both 3D connectivity and partial volume fat correction improved precision from 4.99% to 2.21% test–retest error. Validation against semi-automated methods showed R^2^ from 0.92 to 0.98 with fully-automated ITSA routinely yielding more conservative computations of IMF volumes. Quality control shows 7% of cases requiring manual correction, primarily due to IMF merging with subcutaneous fat. A full workflow described methods to export tags for manual correction.

**Conclusions:**

The greatest challenge in segmenting IMF from MRI is in selecting a dynamic threshold that consistently performs across repeated imaging. Fully-automated ITSA achieved this, demonstrated low short- and long-term precision error, conducive of use within RCTs.

**Supplementary Information:**

The online version contains supplementary material available at 10.1186/s13395-024-00365-z.

## Introduction

Fatty infiltration of skeletal muscle, in the form of INTRA- (within muscle group) and INTER- (between muscle groups) muscular fat (IMF) are associated with metabolic syndrome [1, 2], muscle dystrophy [3], inactivity and aging [4]. The overall accumulation of fat within muscle is also associated with increased risk of hip fractures in older adults [5]. While IMF content varies with physical activity [6], its role in the progression of a variety of diseases is a potential target for therapies such as exercise or pharmacologic interventions. However, studies that measure the success of such interventions requires an IMF metric that is sensitive to differences both between individuals at a given timepoint and within individuals over time. This requires high precision and repeatable measurement, which are dependent on both acquisition and segmentation.

MRI is a well-established modality for imaging IMF, achievable on simple T1-weighted images commonly obtained as a part of standard of care, even in the absence of fat–water separation (Dixon) [7, 8]. While Dixon imaging can rule out potential water accumulation in T1-weighted images, the prevalence of muscle edema in the general population is likely low given its primary causes are related to the presence of inflammation, infarction, lacerations, sports injuries, compartment syndrome, and myopathies [9]. Existent automated IMF segmentation algorithms for either T1-weighted or Dixon sequences have primarily segmented larger streaks of INTER-muscular fat, but relegate finer streaks of INTRA-muscular fat to a sub-property of muscle, often due to the lack of algorithm sensitivity [10, 11]. Other investigators were successful in segmenting INTRA-muscular fat but due to the larger pixel sizes used (> 1.0 mm), the amount captured was limited [12, 13]. Current methods also fail to describe a workflow describing the inevitable need to correct mislabeled masks. Manual correction of boundaries is often necessary in individuals with abnormally large amounts of fat, fat heterogeneously partitioned into one muscle group (ie. muscle dystrophy), or cases where fat is virtually absent (athletes) [14, 15].

Deep learning methods have primarily focused on segmentation of muscle groups followed by fat computation using traditional algorithms [16, 17]. Only two studies applied deep learning directly to IMF segmentation – one convolutional neural network that outputs actual segmentations [18]; and another that only yields Goutallier semi-quantitative scores [19]. The former method succeeded with a DICE coefficient of 80.1% but was based on a small group of 50 healthier individuals, again without describing situations meriting manual correction. Given the need for a large amount of training data, the future success of deep learning methods could benefit from accelerated generation of ground truth labeling using more traditional algorithms. Furthermore, with progressive success of fully-automated IMF segmentation methods in MRI, open access algorithms will enable the customizations necessary for integration into radiology information systems.

High resolution (< 1.000 mm pixel size) MRI scans can display fine streaks of IMF within muscle groups. However, previously reported fuzzy cluster muscle and IMF segmentation methods may not be sufficiently calibrated to detect these thinner geometries [12]. Simple thresholding algorithms can categorize individual pixels into muscle or IMF based on bimodal histograms of pixel intensities, but consistent threshold selection remains unreliable due to signal variability between/within scanners and between/within slices [20]. This limitation is largely a factor of coil and magnet gradient variability, and makes it difficult to standardize or calibrate MR images. Fat within Dixon images have been labeled as IMF based on 50% fat fraction thresholds [21, 22], but this method also misses (partial volumed) thinner streaks of INTRA-muscular fat, especially in healthy individuals.

**Fig. 1:**
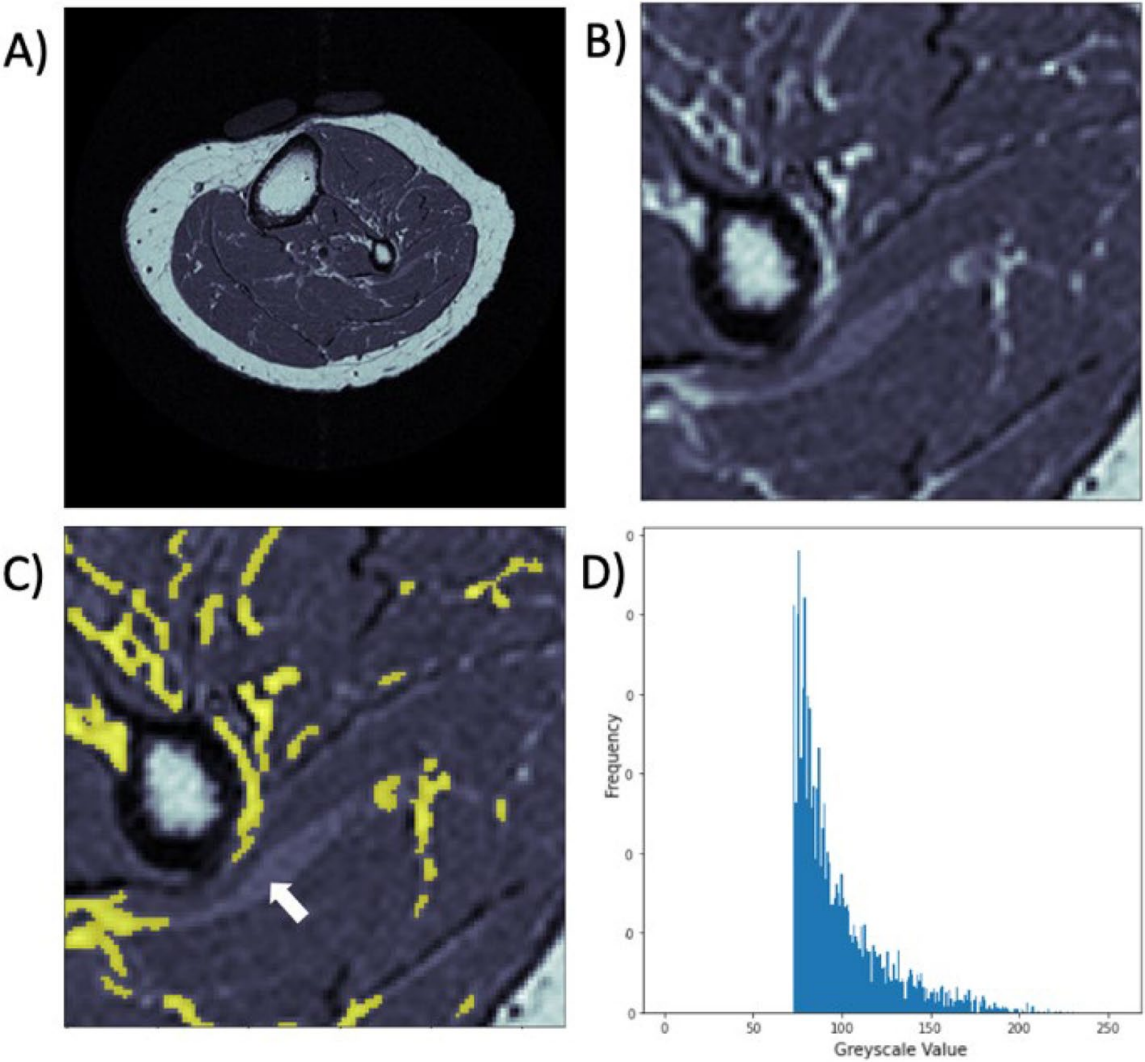
Sources of precision error in the previously-validated semi-automatic application of the ITSA [14]. (**A**) An example of a raw axial FSE MR slice in the mid-calf. (**B**) Zoomed in view of A showing distribution of brighter and fainter (partial-volumed) voxels of fat. (**C**) Successful IMF segmentation applied by ITSA (in yellow), showing remaining voxels of fat that could be quantified. The white arrow points to remaining fat that was not captured due to its greyscale values being below the final threshold identified by ITSA. (**D**) Histogram showing distribution of fat pixel signal intensities

To address the issue of threshold selection amidst the challenge of signal variability, we previously designed and validated a method that automatically optimized slice-specific threshold selection in an iterative fashion [14]. The key features included initial seed threshold identification by histogram shapes, island removal steps to limit the influence of noise, computation of a new threshold based on segregated tissue features, and iteration until convergence (iterative threshold-seeking algorithm (ITSA)) [14]. Despite passing the benchmark for precision error (i.e. root mean square coefficient of variation (RMSCV) < 5%), human error was introduced by manually delineating the muscle fascia for subcutaneous fat separation. There remain partial volumed voxels of fat not factored into the fat volume calculation (Fig. 1C). Further, pruned islands of bright signals labelled as noise may belong to ends of larger fat streaks in the Z dimension.

In the present study, we therefore aimed to 1) fully-automate ITSA with fascial delineation, Z-connectivity check, and fat partial volume correction applied within an open-source Python environment; 2) describe its reliability within short- and long-term test–retest calf images; 3) demonstrate its versatility of application to calf and thigh MRI scans; and 4) describe the workflow necessary to correct imperfections and streamline data outputs.

## Methods

### Overall study design

This was a methodological study evaluating the precision of IMF metrics derived from the fully-automated ITSA algorithm, applied to short- (within day) and long-term (1-year) test–retest MRI calf scans derived from a subset of Hamilton site participants of the Canadian Multicentre Osteoporosis Study (CaMos) [23]; and assessing clinical correlations in a cross-sectional analysis across two cohorts: the Appendicular Muscle and Bone Extension Research Study (AMBERS, using calf MRI scans) [24] and the Osteoarthritis Initiative (OAI, using thigh MRI scans) [25].

### CaMos Hamilton subcohort methods

#### Selection

This study subcohort was a random selection of 57 community-dwelling women 60–85 years of age from the Hamilton, ON chapter (*N* = 1068) of the CaMos Study (*N* = 10,424) who completed FSE MRI scans at the mid-calf (*N* = 98). Further details of the sampling framework were described previously [23, 26]. Participants were excluded if their weight exceeded 250 lbs and if they had any contraindications to MRI.

#### MRI scans

At baseline, all participants completed two sequential MRI scans with repositioning on the same day with sequences prescribed at the 66% site of the non-dominant leg (as defined by the leg that is not often used to kick a ball). Among the 57 participants, 38 completed a follow-up MRI 1 year later at the same site. Imaging Parameters: FSE images without fat–water separation were collected on a 1.0 T peripheral(p) MRI (OrthOne, GEHealthcare) using a 180 mm knee coil, yielding 10 transaxial contiguous 0.312 × 0.312 mm in-plane pixel size slices at 2.0 mm thickness (TR/TE = 600/23 ms, NEX = 3, echoes = 1, flip angle = 40°, and bandwidth = 25 kHz). The centre 8 slices were used in all analyses due to signal loss at end slices. Full image acquisition details were reported previously [23]. MRIs were excluded if they appeared to exhibit motion artifacts of grade 2 or higher according to a previously established classification atlas [27].

### AMBERS cohort methods

#### Selection

AMBERS recruited 312 postmenopausal women ages 60–85 from the Hamilton, ON chapter of the Global Longitudinal Study of Osteoporosis in Women (GLOW), which was a worldwide prospective cohort study on fracture risk [28]. Participants were excluded if their weight exceeded 250 lbs and if they had any contraindications to MRI.

#### MRI scans

Of 312 participants, 296 completed MRI mid-calf scans of the non-dominant leg (defined similarly as above) using the same 1.0 T magnet and FSE MRI protocol as described in the CaMos subcohort above.

### OAI cohort methods

#### Selection

The OAI is a multi-centre, longitudinal, observational study focusing primarily on knee osteoarthritis (OA) among 4796 men and women (58%) 45 to 79 years of age. Specific inclusion and exclusion criteria for the incidence and progression cohorts were described previously – this includes contraindications to MRI [25]. The data and images used are publicly available at https://nda.nih.gov/oai/. The analysis performed here used 105 randomly-selected participants' MRI thigh images (3.E.1 and 3.C.2) and data from the V03 (24 month) visit.

#### MRI scans

A 3.0 T Siemens Magnetom Trio MRI scanner acquired bilateral thigh scans for all participants selected. Imaging parameters: Axial T1-weighted turbo spin echo images were obtained from the quadriceps region centered at 100 mm above the distal femoral epiphysis, yielding 15 slices each 5.0 mm thick with in-plane resolution of 0.977 × 0.977 mm (TR/TE: 500/10 ms, 500 mm FOV, 0 mm gap). All 15 slices were used in analyses. Further details of imaging parameters were previously reported [29].

### Modifications to semi-automated ITSA algorithm

#### Pre-processing and automated region of interest (ROI) delineation

Prior to analyses, all MR images were submitted through the Contrast Limited Adaptive Histogram Equalization (CLAHE) procedure [30] to correct for radiofrequency field (B1) inhomogeneity in the image, an artifact that is commonly observed in T1 or proton-density-weighted images (Figure 3). Previously, the muscle ROI was identified by manual contouring of the fascial border and cortical bone outlines [14]. Here, we automated this process by applying a multi-Otsu algorithm which highlighted both subcutaneous and marrow fat regions, generating a mask from which we then derived muscle, marrow and bone segmentations.

#### Bone ROI

From the initial multi-Otsu filtered mask, only low signal intensity objects above 234 mm^2^ in area representing cortices were kept, as determined empirically. Tibia and femora were identified by summating fat signals across all slices. It was presumed that the high marrow fat within long bone is the only structure co-aligned axially to yield the highest summated value across slices. These regions were used as seed points to expand into fat regions coinciding with bone. The final bone marrow regions were merged with the low signal intensity mask containing the cortices.

#### Muscle ROI

A rough muscle mask was generated from the multi-Otsu filtered mask by removing small objects and holes below size 60cm^2^ and 180cm^2^ respectively – as determined empirically, followed by morphological closing procedures. The inverse of the image, representing only the muscle region without subcutaneous fat, was then flood-filled and subjected to an outer contour search, the result being the seed input muscle contour. This irregularly-shaped seed muscle contour (following the edges of the muscles) was refined using a convex hull to better capture the fascial boundary, which usually displays as a smooth elliptical shape, and envelopes INTER-muscular fat. The resultant elliptical contour was further refined using three rounds of a snake algorithm (from course to fine adjustment) with empirically-defined sets of smoothing parameters: contraction speed, smoothness, and attraction.

**Fig. 2:**
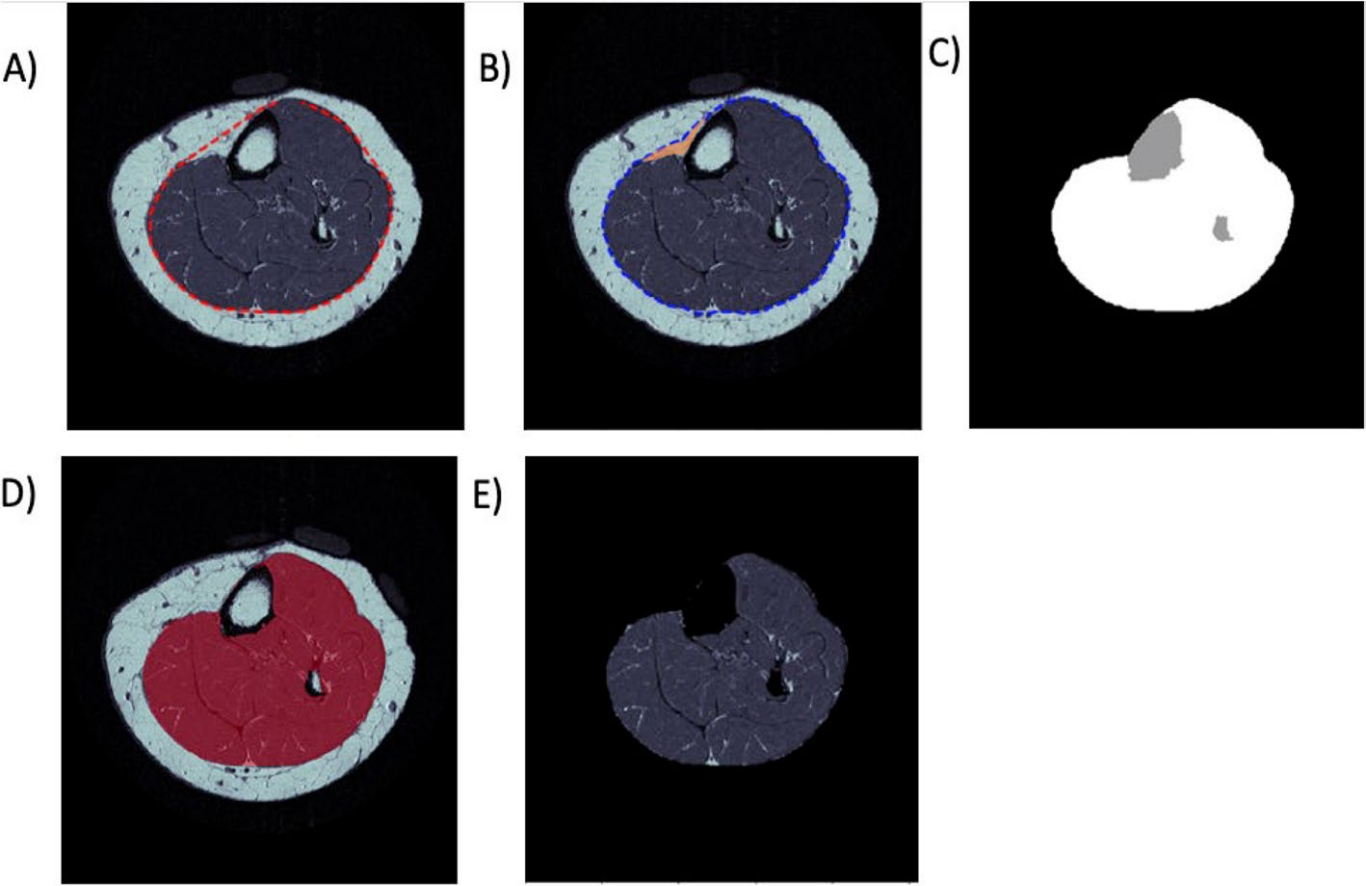
Automation of muscle-fat ROI on which ITSA was applied. (**A**) The Otsu algorithm was used to estimate a threshold that can be used to generate a binary mask of the muscle border. The OpenCV contours function is used to determine the coordinates of the estimated muscle border, represented by the red dashed line. (**B**) Snake algorithm is used to adjust and improve accuracy of the muscle border coordinates, represented by the blue dashed line. Mistakenly included a piece of subcutaneous fat in contact with the tibia, and marked for removal is in orange. (**C**) Bone, represented by grey, is segmented by expanding a seed point identified by maximum summated marrow signal across slices, merged with a void signal mask containing cortices and removed from the ROI, represented by white. (**D**) Final muscle mask represented by translucent red is overlaid on top of the raw MRI slice. (**E**) final cropped muscle ROI

For calf muscles, the smoothness parameters required to generate a tight fit of the snake against the fascial boundary resulted in the inclusion of more subcutaneous fat outside of the fascial boundary, especially anterior to the tibia (Fig. 2A). These erroneously included segments of subcutaneous fat (overshooting) were removed from the muscle mask by applying an Otsu-determined threshold to the present muscle mask and removing large objects that 1) represented fat, and 2) are in contact with the tibia as defined in the bone segmentation section below (Fig. 2B orange segmentation). To ensure fidelity of these removed objects, the candidate fat marked for removal was compared across all other MR slices to ensure 3D connectivity. Any segments of fat identified through this process that were not connected to other candidate fat within neighbouring slices were not removed.

All of the bone ROIs were subtracted from the muscle ROI, to yield the final muscle ROI.

#### Final mask for ITSA application

**Fig. 3:**
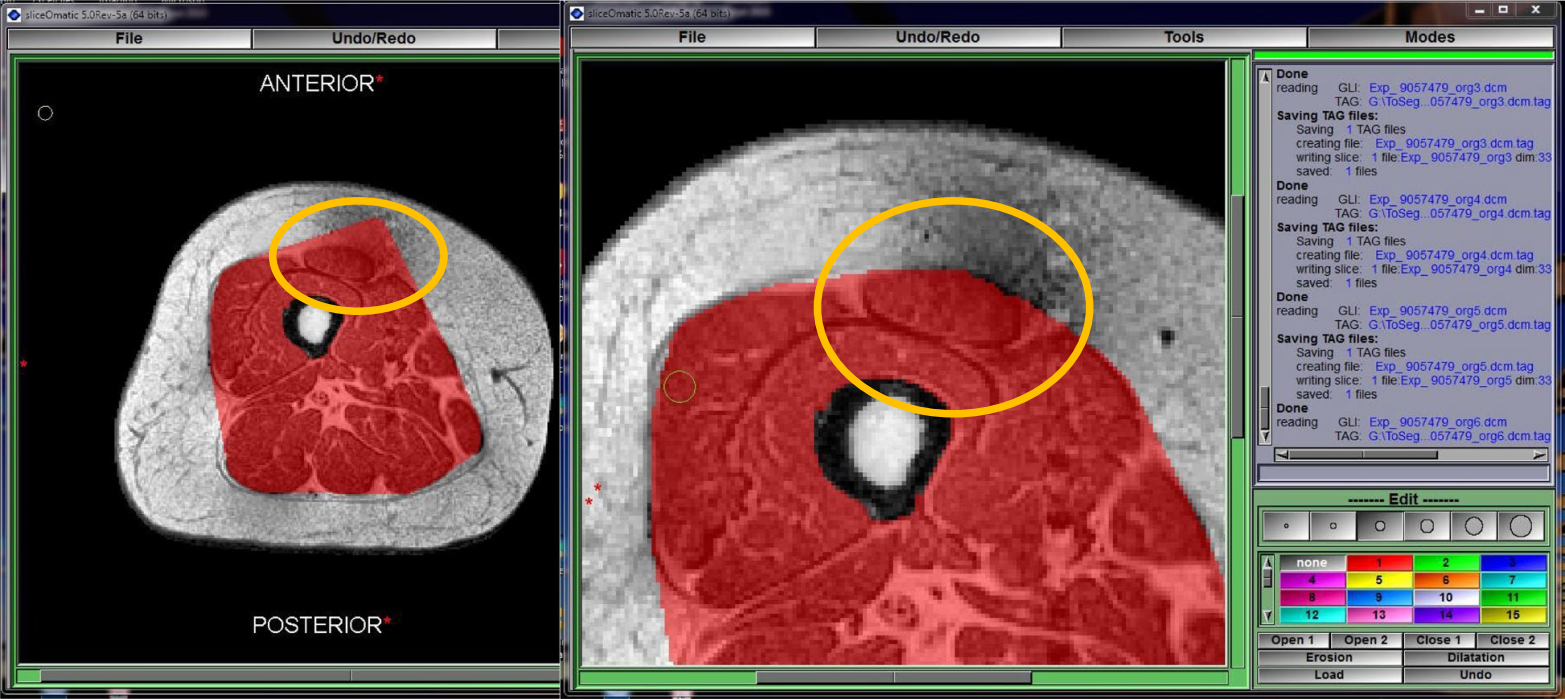
Exported tags for manual correction within Sliceomatic software

To generate the ROI on which ITSA was applied, we multiplied the binary muscle ROI mask by the original raw image (yielding Fig. 2E). It is important to recognize that atypical spatial distributions of muscle and fat, such as extreme cases seen in more diseased and older individuals, may have a higher risk of resulting in unavoidable segmentation errors and will require manual correction. Any outstanding errors in fascial contours in the automated ROIs were labeled as overshooting, undershooting, or overall incorrect. These cases marked for correction were then exported as tag files (tag creation and tag reading scripts integrated within Python notebook) and manually corrected within an interactive software with a GUI containing a set of manual, watershed, adaptive thresholding and region-growing tools (Sliceomatic) (Fig. 3). This included regions where muscle was not captured or where subcutaneous fat was mistakenly included within the muscle ROI mask. Corrected Sliceomatic tag files were automatically read by the algorithm for final fat and muscle feature computation.

#### Addressing partial volumed fat voxels

The ITSA equation (Eq. 1) uses the final mask described above to identify an initial Otsu signal threshold as a seed point to iteratively converge on a revised signal threshold (ST_R_) based on the current mean pixel signal intensity of segmented muscle (SM_i_) and fat (SF_i_), until the previous iteration's ST_R_ is no longer different from the present [14].

1 $${ST}_{R}=\left(1+\frac{{SF}_{i}-{SM}_{i}}{{SF}_{i}}\right)\cdot {SM}_{i}$$

**Fig. 4:**
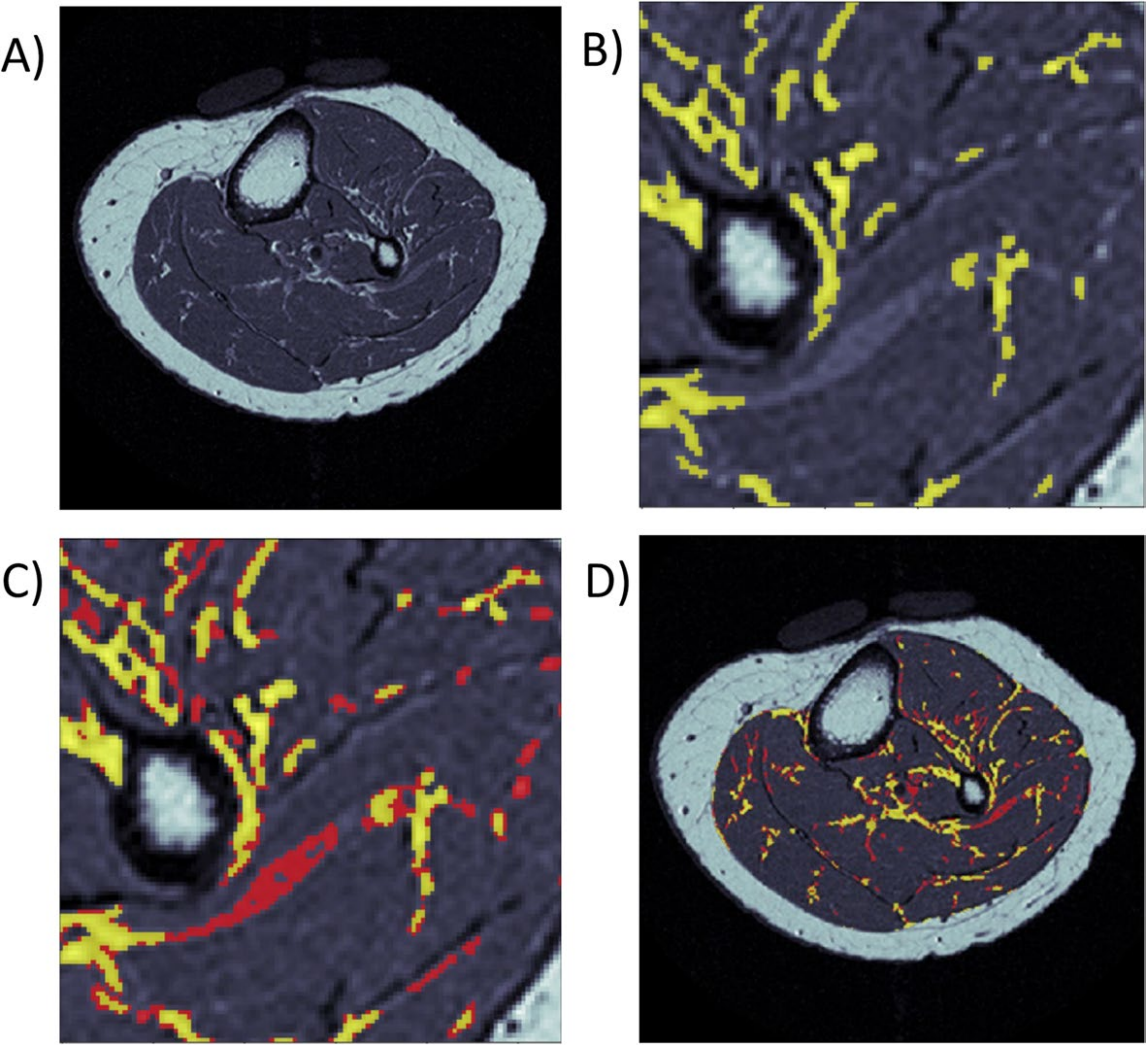
Representative image of partial volumed voxels of fat meriting a second round of ITSA. (**A**) Original MR image. (**B**) Yellow showing IMF segmentation result from applying a first round of ITSA, and remaining light grey areas suggesting outstanding partial volumed fat voxels. (**C**) Red showing IMF segmentation result from applying a second round of ITSA. (**D**) Final segmentation in full view

The first application of ITSA may not be adequately sensitive to segment partial volumed voxels of fat. To address this issue, any potential outstanding voxels of partial volumed fat were collected by subtracting fat from the first round of ITSA to generate a modified ROI on which ITSA was reapplied (Round 2 ITSA). The resultant IMF included fat from both rounds of ITSA (Fig. 4). This solution comes with two trade-offs that must be addressed within the second round of ITSA to optimize measurement accuracy: 1) a lower threshold will increase the risk of falsely segmenting noise as fat, especially in the absence of any substantial fat distribution; and 2) the greyscale value of each presumed fat voxel may actually represent some combination of muscle and fat. Without correcting for signal intensity of fat voxels from the second round of ITSA, any variations in noise and partial voluming could lead to higher test–retest precision error. Another issue that is inherent to ITSA is its necessity of pruning small islands to advance the threshold throughout each iteration, which may remove not only noise, but actual fat that could represent part of a continuous streak in the z dimension.

To address the issue of falsely segmenting noise as fat, or removing fat as noise, we proposed a 3D connectivity solution that checks whether potential voxels of fat are in fact connected in the z dimensions. To resolve the issue of heterogeneous quantities of fat across partial volumed voxels, we proposed a correction factor to compute fat fraction of partial volumed fat voxels. These solutions are described below:

##### 3D (Z-axis) connectivity check

**Fig. 5:**
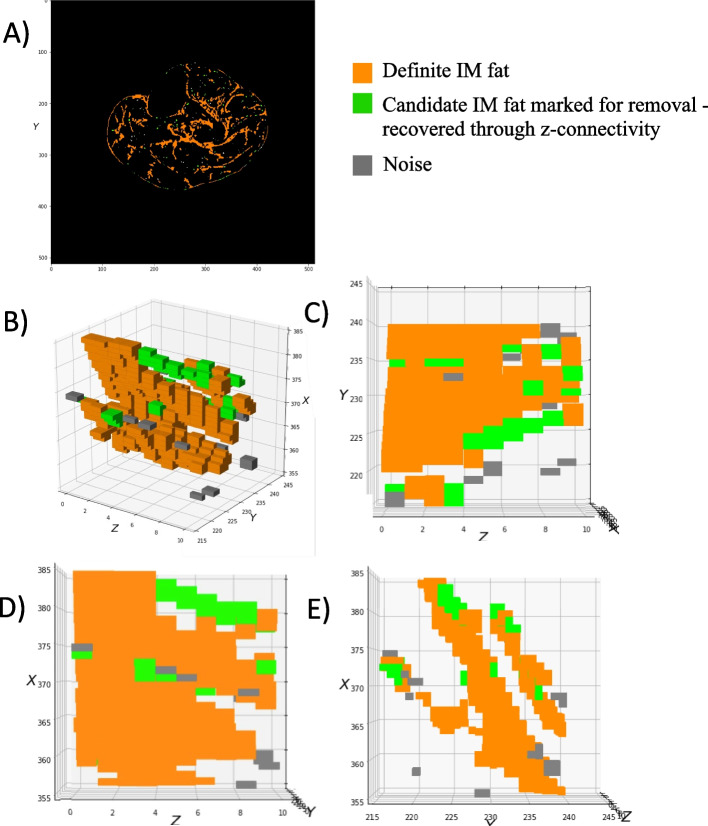
Reclassification of islands of IMF voxels. (**A**) Single slice view of IMF segmentation from round 1 and 2 of ITSA. (**B**) 3-dimensional plot of fat voxels within a sample cropped 30 × 30 × 10 matrix. Orange depicts definite fat, > = 16 mm^2^ in the X–Y plane. Grey depicts unconnected noise, < 8 voxels. Green depicts islands of fat < 16 mm^2^ but connected to fat across adjacent slice(s). (**C**-**E**) orthogonal views of 3D model in the X–Y, X–Z, and Z-Y planes, respectively

For both rounds of ITSA, binary IMF masks were first generated without island pruning for each slice. Groups of fat voxels in the XY plane > = 16 mm^2^ were immediately deemed as IMF (orange, Fig. 5). Groups < 16 mm^2^ but which had connections to fat voxels in any neighbouring slice(s) was retained as fat (green, Fig. 5). All other pixels were excluded from the final IMF segmentation (grey, Fig. 5).

##### Correction for partial volumed voxels

It was assumed that voxels within the IMF segmentation consist of only fat or a combination of muscle and fat; and the continuum of signal intensity between pure muscle and pure fat scales linearly with the percentage of encompassed muscle and fat per voxel according to proton density. A correction factor (C) was generated by comparing the mean intensity of voxels over an entire image volume that contain virtually only fat (SF), only muscle (SM), and some partial volume of both (SV). This correction factor represents the percentage of the volume occupied by IMF within a partial volumed voxel; and its complement (1-C) represents the percentage of volume occupied by muscle within the partial volumed voxel. Therefore, the partial volumed voxel (SV) could be represented by the summation described in Eq. 2a. A rearrangement of this equation to isolate C yields Eq. 2b.

2a $$S{F}_{max}\cdot C+S{M}_{x}\cdot (1-C)=S{V}_{x}$$

2b $$C=\frac{S{V}_{x}-S{M}_{x}}{S{F}_{max}-S{M}_{x}}$$

Where:

SF_max_ is defined as the highest quartile of signal intensity from IMF voxels from the first round of ITSA (as a sensitivity analysis, the mean subcutaneous fat signal was also considered);

SM_x_ is the mean signal intensity of the muscle mask after subtraction of the entire IMF segmentation;

SV_x_ is the mean signal intensity of the IMF mask voxel in question, from round 1 and 2 of ITSA, with values that are < SF_max_.

#### Muscle and IMF metric computations

The correction factor C was applied to IMF segmentations on a per voxel basis. Total IMF volume was calculated as the sum of full fat voxels (NF) and sum of partial volumed voxels (NV) multiplied by C (IMF_V_ = (NF + NV*C)*VoxelVolume). Total muscle volume was calculated as the sum of full muscle voxels (NM) and the number of partial volumed voxels (NV) multiplied by (1-C) (M_V_ = (NM + NV*(1-C))*VoxelVolume). All voxel sums were multiplied by the appropriate voxel volumes to yield volumes in mm^3^ which were converted to cm^3^ where appropriate. Since the number of slices for calf and thigh scans were dissimilar, the average volumes were calculated across all slices analyzed. The final metrics included IMF volume, muscle volume, and IMF% computed as IMF volume/(IMF + Muscle volume) × 100%.

All image analyses were performed on Python 3.9.15 using Jupyter Notebook (full list of dependencies and environment export available in Appendices) installed on a PC with 6 core × 2.20Ghz CPU, 16.0 GB RAM, and 1.0 GB NVIDIA GeForce GTX 1070 GPU. A flow chart of the algorithm is shown in Supplemental Fig. 1.

### Statistical analyses

Test–retest reliability of IMF, %IMF, muscle volume, and subcutaneous fat volume was evaluated on the 57 same-day repeated MRI scans using root-mean square (RMS) coefficients of variation (CV) (benchmark: < 5%), RMS standard deviation (SD), and a type(2,1)-intraclass correlation coefficients (ICC) as per International Society for Clinical Densitometry (ISCD) guidelines [31]. Long-term precision over 1 year was also measured from the available 38 follow-up MRIs relative to the first of the baseline scans from participants using RMSCV, RMSSD, and least significant change (LSC) computed as 1.96*√2*RMSSD to reflect the minimal change required to detect clinically meaningful difference according to ISCD standards. Bland–Altman analyses were conducted to illustrate differences and relative biases of the present automated method compared to the previously applied semi-automated method [14], as evaluated over the range of possible IMF values. The 95% limits of agreement were computed for each case. To evaluate internal consistency between the semi-automated and the fully-automated measurements, univariable linear regression coefficients and intercepts were computed. These sample sizes used fulfill the 30 degrees of freedom required for precision and validity analyses reported previously [31]. External applicability of the algorithm was evaluated on AMBERS and OAI cohorts, reporting distributional properties of the IMF and muscle outputs; quality of image segmentations were also evaluated by visual inspection given the lack of a ground truth.

## Results

### Cohort characteristics

Both Hamilton CaMos and AMBERS cohorts were between 5–8 years older than the OAI cohort, but each similarly had a predominance of overweight individuals (Tables 1, 2 and 3). The distribution of IMF within calves were similar between CaMos and AMBERS (7.68 ± 4.03% versus 7.94 ± 2.85%), whereas thigh IMF% measured from OAI was more than twice as high (17.11 ± 6.18%) compared to calf IMF% in both CaMos and AMBERS cohorts. Upon visual inspection of the quality of segmentations, both calf and thigh images from AMBERS and OAI cohorts showed high fidelity of IMF, muscle, and subcutaneous fat segmentations (Supplemental Fig. 2).

**Table 1:** Descriptive statistics, muscle and IMF distributions for CaMos cohort

Variable	N		Mean	SD	Minimum	Maximum
Age (yrs)	54		72.1	8.5	59.0	89.0
BMI (kg/m^2^)	54		27.22	4.78	20.32	39.56
Calf IMF volume (cm^3^)	54		8.08	4.51	3.35	26.55
Calf Muscle volume (cm^3^)	54		97.66	15.81	65.46	133.02
Calf IMF% (%)	54		7.68	4.03	3.24	23.57
Variable & Levels	**Total N**	**Freq**	**%**			
Sex						
Female	54	54	100.0			
Male	54	0	0.0			
Race						
Caucasian	54	54	100.0			
Other	54	0	0.0

**Table 2:** Descriptive statistics, muscle and IMF distributions for AMBERS cohort

Variable	N		Mean	SD	Minimum	Maximum
Age (yrs)	280		75.2	5.9	63.0	89.0
BMI (kg/m^2^)	280		29.37	5.56	16.41	48.24
Calf IMF volume (cm^3^)	280		7.41	3.11	2.77	21.86
Calf Muscle volume (cm^3^)	280		85.78	14.72	30.91	128.45
Calf IMF% (%)	280		7.94	2.85	3.40	18.41
Variable & Levels	**Total N**	**Freq**	**%**			
Sex						
Female	280	280	100.0			
Male	280	0	0.0			
Race						
Caucasian	280	279	99.6			
Black	280	1	0.4			
Not at all	279	98	35.1

**Table 3:** Descriptive statistics, muscle and IMF distributions for OAI cohort

Variable	N		Mean	SD	Minimum	Maximum
Age (yrs)	105		67.42	8.13	47.00	80.00
BMI (kg/m2)	105		27.59	4.17	18.00	38.40
Thigh IMF volume (cm^3^)	105		7.85	2.82	2.63	20.15
Thigh Muscle volume (cm^3^)	105		47.20	11.40	23.77	73.04
Thigh IMF% (%)	105		17.11	6.18	6.41	46.67
Variable & Levels	**Total N**	**Freq**	**%**			
Sex						
Female	105	57	54.3			
Male	105	48	45.7			
Race						
Caucasian	105	87	82.9			
Black	105	13	12.4			
Hispanic	105	3	2.9			
Others	105	2	1.9			

### Performance and reliability of fully-automated ITSA with modifications

**Fig. 6:**
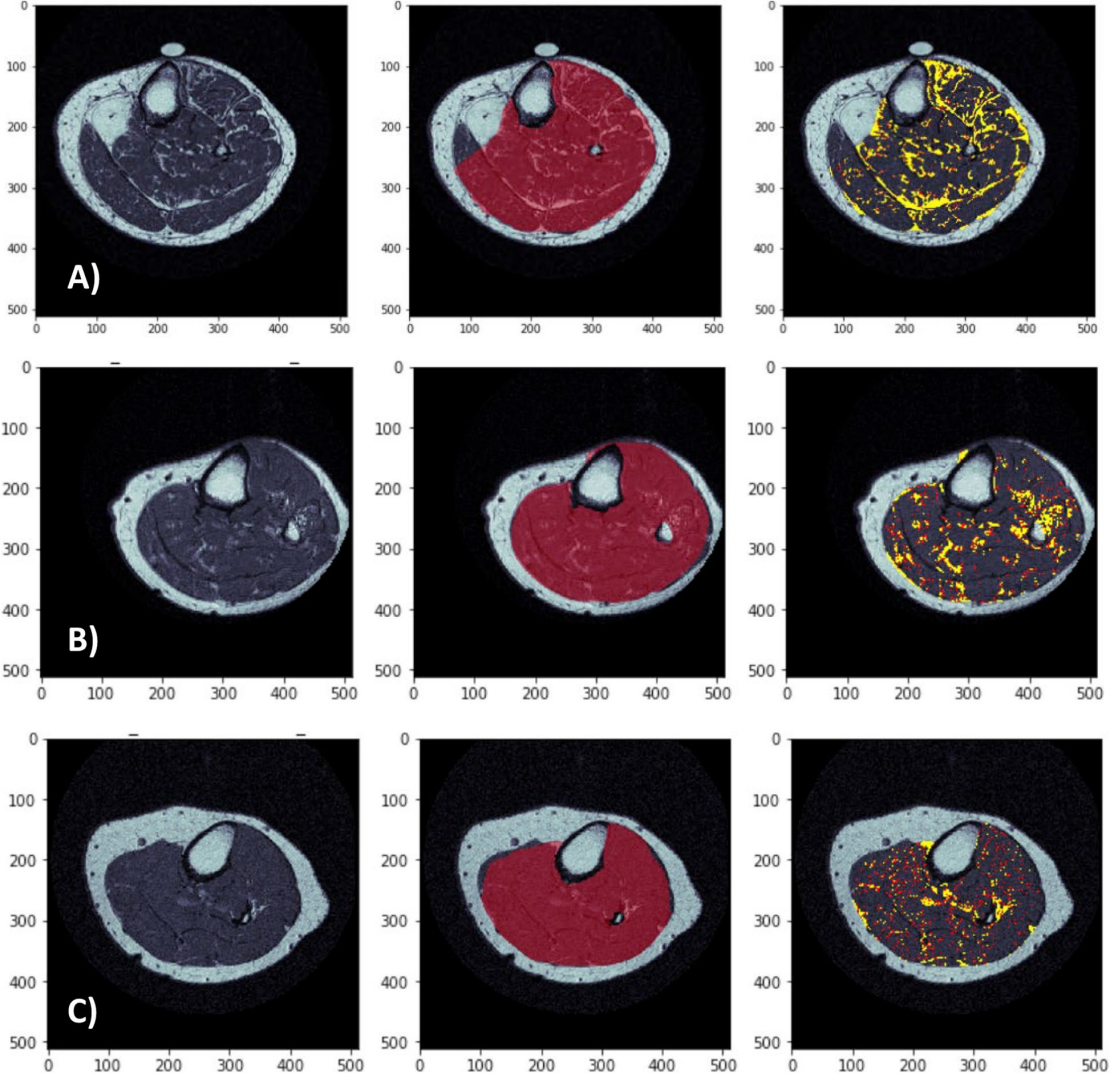
Examples of (**A**) IMF merging with subcutaneous fat, (**B**) motion streaks over-segmenting fat, and (**C**) noise within lean muscle segmented as fat

This improved version of ITSA completed the analysis of a 15-slice stack of T1-weighted MR images in 3.36 ± 0.18 s on an entry-level dedicated graphics PC. This excels in efficiency compared to the semi-automated method (10–15 min). With respect to automating ROI segmentations across the test, retest, and follow-up MRIs from the Hamilton CaMos cohort, 55/57 (96.49%), 55/57 (96.49%), and 33/35 (94.29%) of ROIs, respectively, were deemed successful by visual inspection – the remainder requiring manual corrections by Sliceomatic. A summary of error types is described in Table 4 and illustrated in Fig. 6.

**Table 4:** Summary of systematic errors in automated segmentation

Error types	Test (freq)	Retest (freq)	Follow-up (freq)	Total (freq, %)
1) IMF merging with subcutaneous fat	2	2	2	6/92 = 6.5%
2) Motion streaks mistaken as fat	1	2	1	4/92 = 4.3%
3) Noise within lean muscle segmented as fat	1	0	0	1/92 = 1.1%
Total (freq, %)	4/57 = 7.0%	4/57 = 7.0%	3/35 = 8.6%	11/92 = 12.0%

Frequencies of three error types are described: 1) IMF mistakenly labeled as subcutaneous fat due to poor discernibility of fascial boundaries. 2) Motion streaks appearing as fat were mistakenly segmented as IMF. 3) Noise with higher signal intensity within an otherwise lean muscle was erroneously segmented as IMF since ITSA searches for a bimodal distribution of fat and muscle. See Fig. 6 for examples of each

**Fig. 7:**
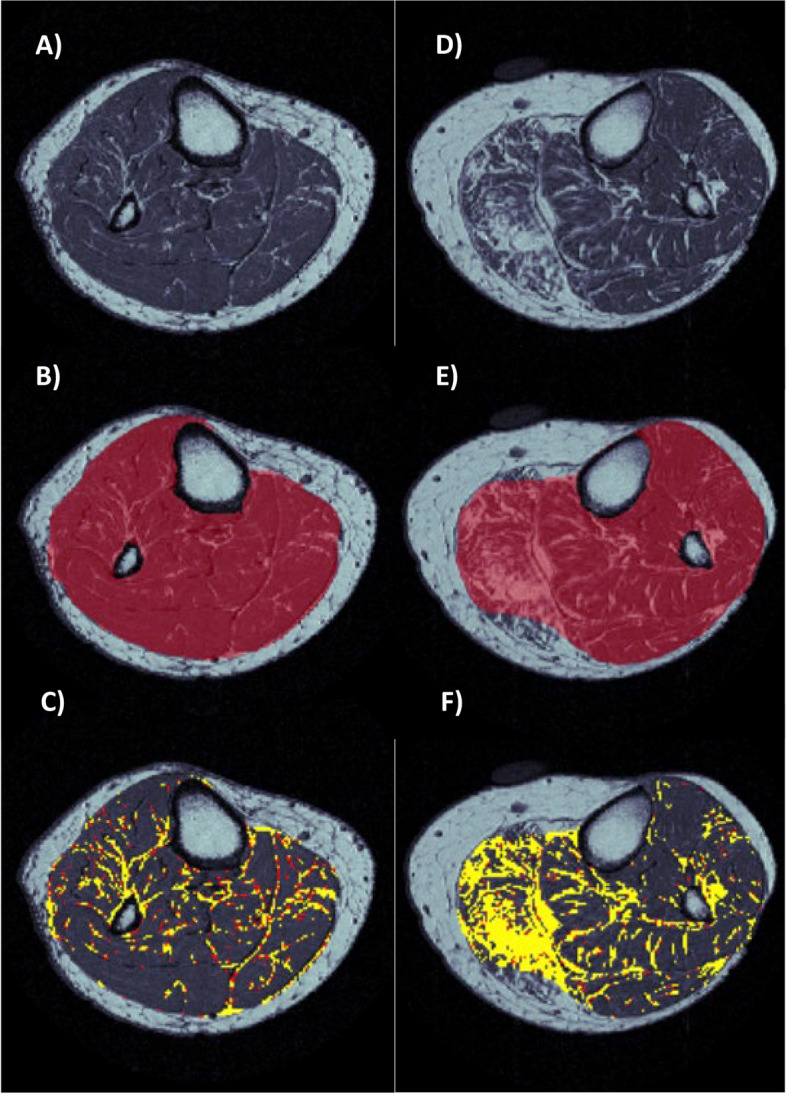
Automation of ITSA IMF segmentation for a prototypical leg (**A**-**C**) versus an individual with muscle dystrophy (**D**-**F**). (**A** & **D**) Original image. (**B** & **E**) Isolation of muscle ROI after filters, snake and convex hull algorithms. (**C** & **F**) Resultant IMF auto-segmentation without corrections displaying yellow as fat identified in round 1 of ITSA and red as partial volumed fat from a second round of ITSA

The first type of error was caused by indistinct fascial boundaries between muscle versus subcutaneous fat resulting in missing parts of the muscle ROI. This was particularly prominent in one individual with evidence of muscle dystrophy (Fig. 7). However, this and similar cases were easily rectified by manual correction using Sliceomatic.

The second type of error resulted from motion artifacts that distorted the representation of IMF within the ROI. Although motion grades 2 and higher were removed from analysis, it is possible that remaining grade 1 motion scans still contributed to overestimation of IMF. These cases can be seen in 4 scans across test–retest and follow-up scans.

The third type of error exists primarily in individuals who inherently have leaner muscles, thus exhibiting an absence of a bimodal distribution when selecting a seed threshold for ITSA. This applies to both first and second (where most fat is already removed) rounds of ITSA. The search for a non-existent fat distribution falsely registered noise as fat, resulting in an overabundance of segmented fat in an otherwise lean individual. To circumvent this challenge, we amended to our algorithm post-hoc to reclassify labeled fat pixels into muscle if their pixel intensities were within 2SD of the original mean muscle pixel intensity in the first round of ITSA.

#### Reliability and validity of fully-automated ITSA

The RMSCV for test–retest calf scans was well within the 5% benchmark (Table 5), indicating excellent short-term precision across all metrics. This was supported by high ICCs above 0.90.

**Table 5:** Indicators of short-term reliability for ITSA-derived muscle and IMF metrics

Variable	RMSCV(%)	RMSSD	LSC	ICC(2,1)
Muscle volume (cm^3^)	1.78	1.805	5.002	0.972 (0.954, 0.984)
IMF volume (cm^3^)	2.68	0.242	0.672	0.993 (0.988, 0.996)
IMF%	2.17	0.177	0.491	0.993 (0.988, 0.996)
Subcutaneous fat (cm^3^)	2.63	1.451	4.023	0.997 (0.994, 0.998)

The corresponding LSCs benchmark the minimum clinically important difference required for future clinical studies. Indeed, the 1-year mean absolute changes just exceeded these LSC values (Table 6), with a corresponding percentage change amounting to 6.1 to 10.5%. Applying the second round of ITSA to capture and correct for partial volumed voxels in general improved the precision by 0.4 to 1.6 percentage points (Table 7). Applying either the correction factor for partial volumed voxels, or the 3D connectivity algorithm to ascertain island removal steps, each improved precision by 1.5 to 2.1 percentage points. Applying both of these steps yielded the best test–retest precision of 2.21%.

**Table 6:** Precision-error seen after 1-year follow up from baseline

Variable	RMSCV(%)	mean% 1-yr change	SD% 1-yr change	mean 1-yr change	SD 1-yr change
Muscle volume (cm^3^)	6.46	6.07	6.17	5.606	6.985
IMF volume (cm^3^)	9.90	10.50	10.30	0.804	1.136
%IMF	7.08	8.03	7.42	0.532	0.641
subcutaneous fat ( cm^3^)	7.79	7.66	7.14	4.411	5.463

**Table 7:** Variations in %RMSCV for %IMF with versus without application of correction factor (CF) and/or 3D connectivity (3D) A) when second round of ITSA was applied to account for partial-volumed voxels versus B) when it was not applied

A)	CF on	CF off
3D on	2.21	2.87
3D off	3.40	4.99
**B)**	**CF on**	**CF off**
3D on	2.61	-
3D off	-	3.39

**Fig. 8:**
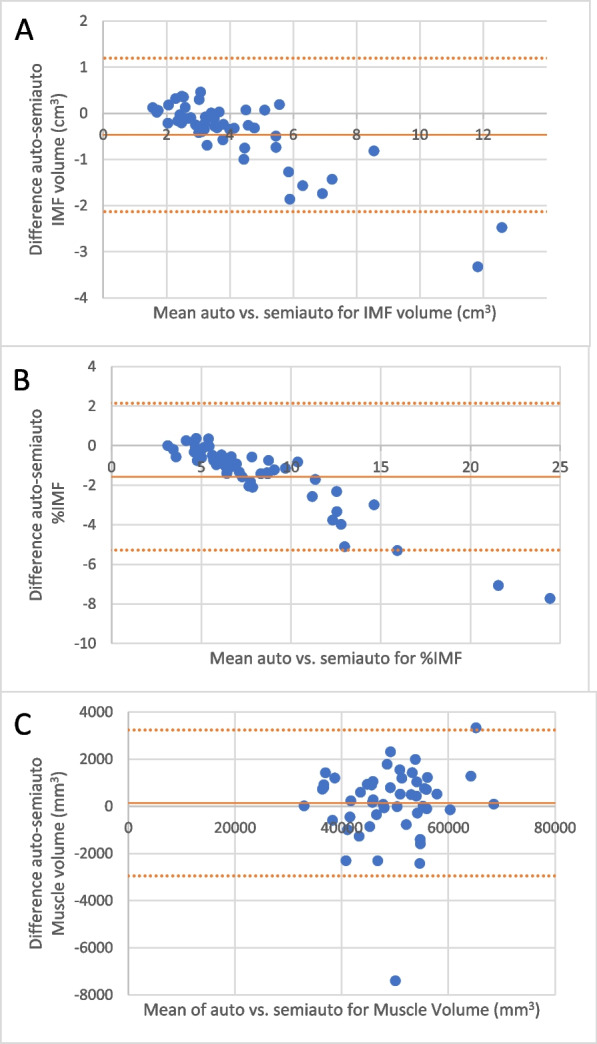
Bland–Altman analysis for differences in (**A**) IMF volume, (**B**) IMF%, and (**C**) Muscle volume metrics between the fully-automated versus semi-automated methods. Red dotted lines indicate 95% limits of agreement. Solid lines indicate mean difference between methods

**Fig. 9:**
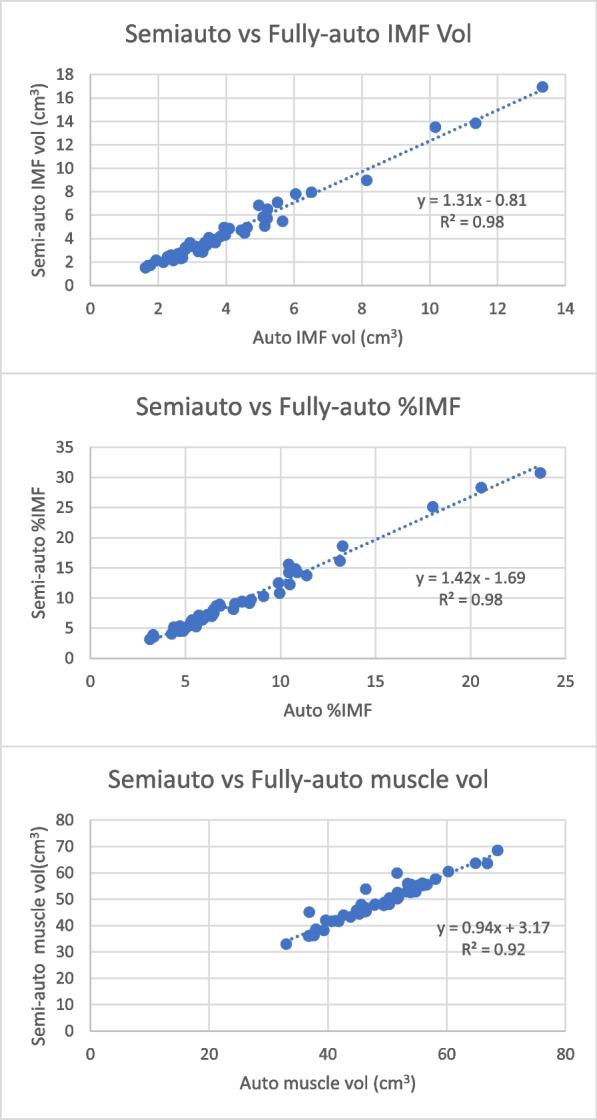
Scatter plots illustrating validity of IMF and muscle volumetric measures. Dotted lines represent univariable fitted regression slopes. Unity (1.0) indicates an ideal fit. Values < 1.0 indicate fully-automated method yielding smaller values than semi-automated method. Values > 1.0 indicate fully-automated methods yielding larger values than semi-automated method

Metric agreement between the fully-automated ITSA and the previously-validated semi-automated method are illustrated in the Bland–Altman plots in Fig. 8. For IMF volume and %IMF, the fully-automated method was more conservative in segmenting IMF, especially for cases where IMF distribution was higher. However, for muscle volume measurements, directional biases were not apparent at any range of values. The systematic relative underestimation of fat but consistent estimation of muscle was also represented in linear regression plots (Fig. 9), though in all cases demonstrating a high R^2^ overall (> 0.90) between the two methods. Therefore, the present automated segmentation method demonstrates high internal consistency, especially given the manual muscle ROI contouring method in the semi-automated method was rigorously confirmed by visual inspection.

## Discussion

### Summary of findings

Here we described a fully-automated muscle boundary delineation and a robust iterative thresholding seeking algorithm (ITSA) with partial volume voxel correction and 3D connectivity checks to segment and quantify muscle volume, IMF, and %IMF with high reliability. We demonstrated its versatility in application to both calf and thigh MR images acquired in axial TSE or FSE sequences, and across three separate cohorts. The methods addressed previous limitations in tedious manual ROI contouring, unsegmented partial volumed voxels, and the need to distinguish between noise versus true fat signals. The success rate of automated muscle ROI delineation was approximately 95% based on visual inspection. We discovered that implementing partial volume voxel correction and a 3D connectivity check each contributed to improving the short-term test–retest precision from just under 5% to 2.21%, a two-fold improvement, even mitigating potential imprecisions resulting from segmentation of partial volumed voxels. One-year changes just exceeded the recommended LSCs indicating appropriate sensitivity for annual longitudinal follow-up. Compared to the previous semi-automated method, the automated IMF volumes showed 30–40% less overestimation.

### Features and performance of automated ITSA versus other algorithms for IMF segmentation

Previously, manual segmentation of IMF yielded unacceptably poor precision from analysis-reanalysis of the same image (RMSCV = 9.02% unblinded, 26.21% blinded), which would be too imprecise for evaluating differences between individuals or changes over time [14]. The semi-automated method vastly improved this using the first edition of ITSA but still required manual muscle and bone ROI delineation, which translated to 10–15 min per analysis of 15 slices, with precision of 3.56 to 4.29% for test–retest and same-image interobserver analyses [14]. The improvements reported here significantly reduces the need for manual intervention at the stage of automated segmentation, thus analysis-reanalysis of the same image either from one or multiple observers, is virtually 0% error upon algorithm output, and accomplished within 5 s for 15 slices. After reviewing the results of algorithm outputs, 6.5% of unavoidable cases required manual correction, and was often the case for more diseased individuals.

#### Threshold selection in T1-weighted images

T1-weighted MRI is by far one of the most common types of acquisition sequences applicable on all MR modalities and tools developed for analysis of its derived images therefore have wide applicability – this includes images acquired beyond musculoskeletal indications. However, the biggest challenge with IMF segmentation on T1-weighted images is identifying the threshold for separating muscle from fat. Ogawa et al. (2017) reviewed various methods for fat-muscle separation on T1-weighted MRI [32–37] and cited outstanding inconsistencies in threshold identification [38], motivating others to develop methods to reproducibly isolate a single threshold. Some investigators generated bimodal histograms by placing ROIs within different sources of muscle and fat [39], others used a histogram mid-point method to average out fat versus muscle signals over multiple slices [40–42]. These methods still yielded poor reproducibility (9.0 to 15.3% short-term RMSCV with rescan and repositioning [42, 43]), especially on test–retest sets of images, and required much user intervention. This limitation has been addressed by the present study using an iterative search approach to converge on a consistent signal threshold between fat and muscle. The critical difference here is that between each iteration, an important small island removal step is applied that is further checked for 3D connectivity to prevent the influence of noise on threshold selection. The error rate of 4–6% upon visual inspection is low, and driven primarily by the challenge of fascial boundary delineation.

#### Fascial boundary identification

The second challenge with isolating the muscle ROI from MR images is in clearly defining what is considered muscle or INTER-muscular fat versus subcutaneous fat. While it may be tempting to base this decision on obvious presence of muscle tissue, this would exclude potential sources of INTER-muscular fat existing between muscle groups within the fascial boundary [13]. The fascia presents itself as a weak edge in contrast against fat and muscle. Orgiu [44] and Positano [12] used a similar snake algorithm as the described method here applied to delineate the fascial boundary for proper muscle ROI identification. However, they only measured INTER-muscular and not INTRA-muscular fat. Chaudry more recently applied the A* algorithm, a type of cost-minimizing function, to perform muscle boundary identification, but this required an interface with a manual editing tool to correct for segmentation errors [45]. The resultant interoperator IMF volume reproducibility (5.8%) was still above our 5% benchmark for multi-user reanalysis of the same image. The present study used a series of contour search, convex hull and snake algorithms to achieve a smooth fit across muscle groups while faithfully retaining INTER-muscular fat. However, this method remains challenged by cases where muscle groups are small and INTER-muscular fat quantities are prominent – and in some cases require manual correction. The ability to automatically separate the subcutaneous fat from muscle and capture INTER-muscular fat using the fascia lata was a major benefit of the improved ITSA algorithm. While Dixon imaging might excel at better separating fat from water signals, its ability to discern the fascia lata is weaker compared to T1-weighted images [46] thus limiting the ability to faithfully capture INTER-muscular fat.

#### Clustering and classification techniques for IMF segmentation

Other investigators applied classification-type techniques to label muscle versus IMF. Valentinitsch [47] et al. used a similar series of subcutaneous fat and muscle mask processing steps as we did in the present study on their Dixon images, but was guided by multi-parametric classification. Indeed, IMF separation is still an important challenge in Dixon imaging [46] due to the potential for partial voluming and noise generated from in and out of phase image subtraction. It also appears that the severity of fat infiltration affects performance of existent IMF separation algorithms in Dixon imaging [48]. We have previously applied an earlier version of our ITSA algorithm on Dixon images with success, further highlighting its versatility, but have not validated results against T1-weighted images [49]. Davis et al. [50] measured shoulder INTRA-muscular fat infiltration on T1-weighted MRI, applying fuzzy c-means cluster segmentation to separate fat from muscle on MIPAV software, with interobserver reproducibility ICCs of 0.947 and 0.951, respectively. They also validated these measures against fat–water separated images (6 point Dixon) with correlation of *r* = 0.955, which was not done in the present study. This approach still yielded some re-analysis error due to manual application in the MIPAV software. Using a similar approach applied to T1-weighted MR images, Lareau-Trudel [13] fully-automated the process with fuzzy clustering, snake fascia delineation, and was successful in 80% of cases, but yielding reproducibility just above what was observed here (interobserver 3.3% and intraobserver 5.6%). However, many similar classification-based methods were still affected by unrectified signal inhomogeneity challenges as identified in published figures and the inability to capture finer streaks of INTRA-muscular fat [13, 50, 51].

#### Deep learning for IMF segmentation

There have been some attempts at using convolutional neural networks to segment fat from MRI scans, primarily of the thigh muscles. Kemnitz et al. developed a UNET architecture CNN to segment INTER-muscular but not INTRA-muscular fat from OAI cohort thighs, showing similar sensitivity to weight loss as manual segmentation methods [51]. Importantly, their images were bias-field corrected which yielded superior performance. However, there was little discussion on potential fascial boundary failures and the need for manual correction. Yao et al. reported using two neural networks, the first to identify fascial boundaries with more fidelity, and a second for tissue classification [52]. While INTER-muscular fat was separated from surrounding tissues, INTRA-muscular fat was also left unexamined. Notably, this method performed well even among patients with muscle dystrophy, which we found to often require manual correction using our methods.

### Precision and internal consistency of automated ITSA

The directional bias patterns observed for larger values of IMF reported here between the fully-automated versus semi-automated ITSA are similar to those previously described between semi-automated versus manual methods [14]. The fact that short-term precision improved by as much as two percentage points after the 3D connectivity check and partial volume correction suggest that these sequential implementations were important features in this improved version of ITSA. While we were only able to test reliability in the calf from available test–retest scans in the Hamilton CaMos cohort, we do not anticipate the precision error to be any worse for thigh measurements, especially given the clearer depiction of the fascial boundary within thigh versus calf images. The LSC values for IMF being within the 1-year observed mean % change gives confidence that our methods can be applied to longitudinal studies to measure changes as early as or even sooner than one year.

### Strengths & limitations

The major strength of this study is that the algorithm was designed to segment both INTRA- and INTER-muscular fat, applies methods to faithfully delineate fascial boundaries, accounts for fat connectivity in 3D space, and adjusts for partial voluming effects. These previous challenges have not been addressed so far and here, it was demonstrated that the solutions yielded improved test–retest precision. Some outstanding limitations include the inability to separate INTER- versus INTRA-muscular fat, the lack of validation against fat–water separated images (though it is not expected that water signals could follow similar fat distribution patterns), and the lack of studies measuring sensitivity to longer term changes in functional outcomes. As previously described in detail, IMF segmentation within T1-weighted images may be challenged by the presence of edema [46]. While Marty and Carlier observed an overall increase in T1 signals after exercise [53], it is unclear if the signal variation is likely to impact T1-contrast, which is required to segment IMF. Dixon imaging could separate these confounding effects, and the ITSA tool could be applied to these images in the future (as we previously demonstrated [49]) rather than relying on a 50% fat fraction threshold that may be less suitable for healthy individuals [46].

## Conclusions

The ITSA method of threshold convergence combined with 3D connectivity verification and partial volumed voxel signal corrections yielded a reliable algorithm for reproducible INTER- and INTRA-muscular fat segmentation from repeated MR images. This technique has shown clear versatility in its application to both thigh and calf muscles. The algorithm is open source and a clear workflow is provided for quality control and manual correction. The precision errors described in this paper also give insight on its ability to measure change beyond 1-year precision error, which can be applied to future sample and power calculations. Simple empirical adjustments could be made to adapt the same algorithm to muscle groups in other appendicular sites.

## Supplementary Information

Supplementary Material 1.

Supplementary Material 2.

## Data Availability

No datasets were generated or analysed during the current study.
