# Supplementary material for: Fully-automated segmentation of muscle and inter-/intra-muscular fat from magnetic resonance images of calves and thighs: an open-source workflow in Python
Source: Skelet Muscle. 2024 Dec 27;14:37. doi: 10.1186/s13395-024-00365-z (PMC11674188; doi:10.1186/s13395-024-00365-z)
Supplement: Supplementary file 1 — Supplementary Material 1. [file 13395_2024_365_MOESM1_ESM.docx]

# Supplemental Table 1: Python libraries used and their purpose

| **Library/Package** | **Purpose** | **Specific Functions Imported** |
| --- | --- | --- |
| tensorflow | Machine learning, deep learning, and neural network models | N/A |
| pandas | Data manipulation and analysis | N/A |
| os | Operating system interface, file and directory management | N/A |
| pydicom | Handling DICOM medical imaging files | N/A |
| cv2 | Computer vision and image processing | N/A |
| matplotlib | Plotting and data visualization | pyplot |
| numpy | Numerical computing and array manipulation | N/A |
| SimpleITK | Medical image analysis | N/A |
| sys | System-specific parameters and functions | N/A |
| skimage | Image processing and computer vision | morphology, segmentation, data, io, filters |
| scipy.ndimage | Provides image processing functionalities, particularly for filtering, morphology, and interpolation of multidimensional arrays. | binary_dilation |
| imutils | Image processing utilities (common helper functions) | N/A |
| typing | Type hinting for function annotations | Any, Dict, Tuple, List |
| Pillow | Image processing and manipulation (fork of the Python Imaging Library) | ImageEnhance, Image |
| math | Mathematical functions | N/A |
| astropy | Astronomy-related computing (specifically QTable for table manipulation) | QTable |
| tabulate | Pretty-print tabular data | N/A |
| statistics | Statistical functions, such as mean | mean |
| ipywidgets | Interactive widgets for Jupyter notebooks | N/A |
| IPython.display | Jupyter-specific display functionalities | display |
| shutil | High-level file operations like copying and removing files | N/A |
