# Supplementary material for: Fully-automated segmentation of muscle and inter-/intra-muscular fat from magnetic resonance images of calves and thighs: an open-source workflow in Python
Source: Skelet Muscle. 2024 Dec 27;14:37. doi: 10.1186/s13395-024-00365-z (PMC11674188; doi:10.1186/s13395-024-00365-z)
Supplement: Supplementary file 2 — Supplementary Material 2. [file 13395_2024_365_MOESM2_ESM.docx]

**Supplemental Figure 1.** Flow chart of algorithm indicating areas where user input is required and where quality assurance (QA) workflows are implemented.

**Supplemental Figure 2. External applicability of ITSA algorithm on calf and thigh images in AMBERS and OAI cohorts, respectively.**

**
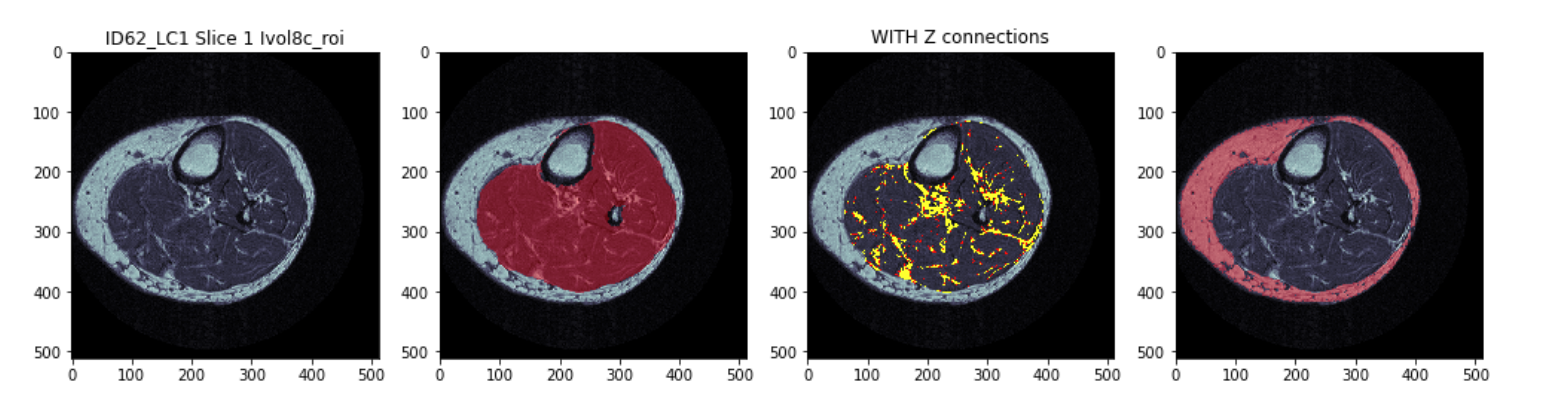
**

**
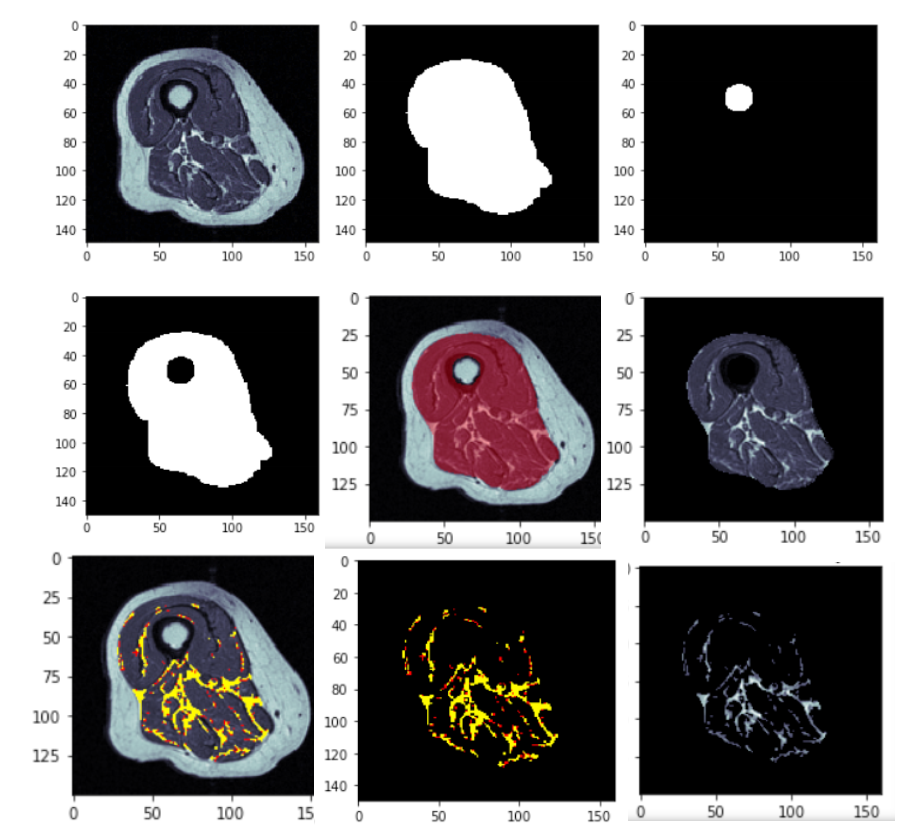

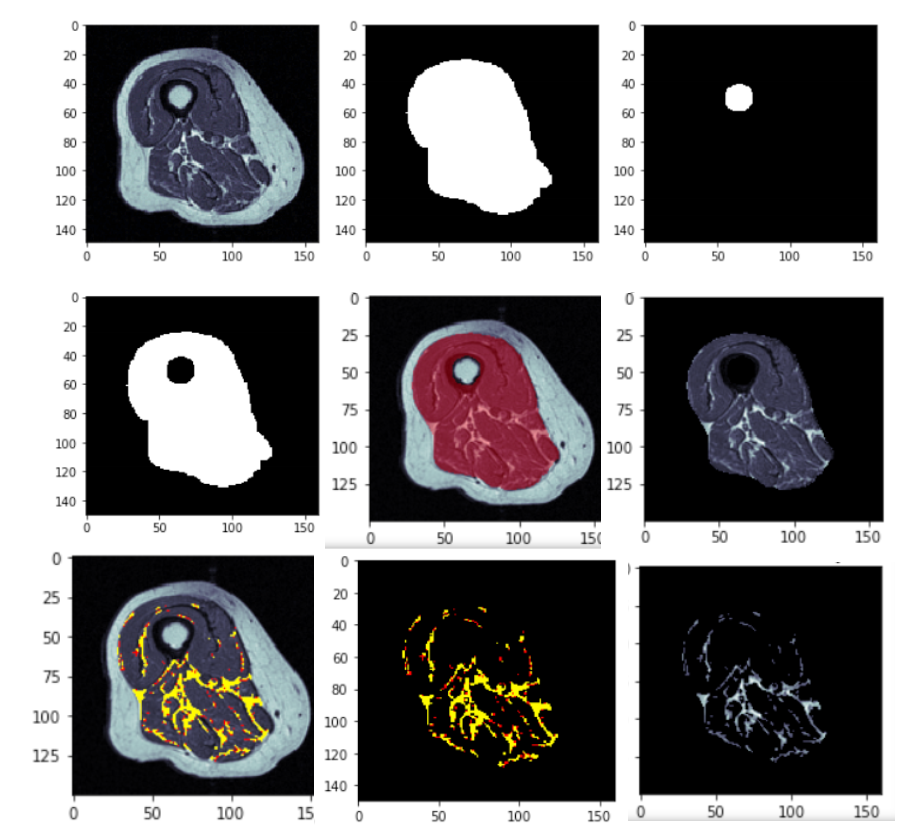

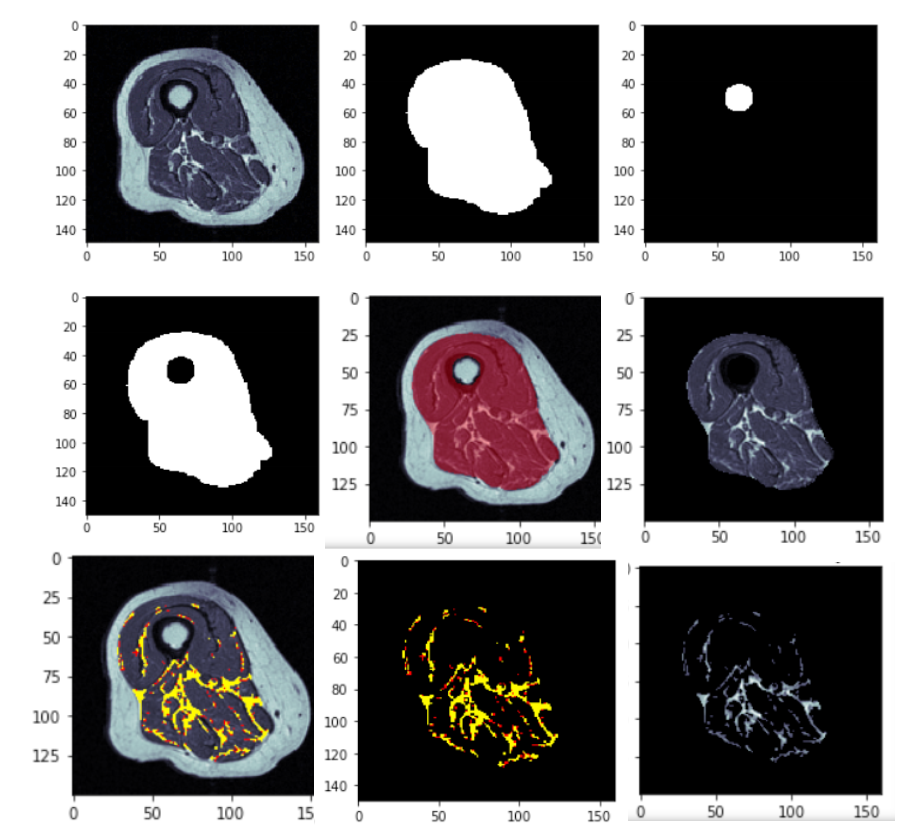
**

**Supplemental information.** Python environment (version 3.9.15) and libraries used. Python library env file available on GitHub.

**List of libraries used:**

import tensorflow as tf

import pandas as pd

import os

import pydicom as dicom

import cv2

import matplotlib.pyplot as plt

import numpy as np

import SimpleITK as sitk

import sys

import skimage

from skimage import morphology

from skimage.segmentation import active_contour

from skimage import data, io, img_as_ubyte,filters

from skimage.filters import threshold_multiotsu

from skimage.measure import label, regionprops

from scipy.ndimage import binary_dilation

import matplotlib as mpl

import imutils

from typing import Any, Dict

from typing import Tuple, List

from PIL import ImageEnhance

from PIL import Image

import math

from astropy.table import QTable

from tabulate import tabulate

from statistics import mean

import ipywidgets as widgets

from IPython.display import display

from ipywidgets import interact, interactive, fixed, interact_manual

from ipywidgets import *

import pydicom

import shutil

**List of technical dependencies:**

absl-py @ [file:///home/conda/feedstock_root/build_artifacts/absl-py_1705494584803/work](file:///\\home\conda\feedstock_root\build_artifacts\absl-py_1705494584803\work)

aiohappyeyeballs @ [file:///home/conda/feedstock_root/build_artifacts/aiohappyeyeballs_1723793936996/work](file:///\\home\conda\feedstock_root\build_artifacts\aiohappyeyeballs_1723793936996\work)

aiohttp @ [file:///D:/bld/aiohttp_1723940726527/work](file:///D:\bld\aiohttp_1723940726527\work)

aiosignal @ [file:///home/conda/feedstock_root/build_artifacts/aiosignal_1667935791922/work](file:///\\home\conda\feedstock_root\build_artifacts\aiosignal_1667935791922\work)

annotated-types @ [file:///home/conda/feedstock_root/build_artifacts/annotated-types_1716290248287/work](file:///\\home\conda\feedstock_root\build_artifacts\annotated-types_1716290248287\work)

anyio @ [file:///home/conda/feedstock_root/build_artifacts/anyio_1717693030552/work](file:///\\home\conda\feedstock_root\build_artifacts\anyio_1717693030552\work)

argon2-cffi @ [file:///home/conda/feedstock_root/build_artifacts/argon2-cffi_1692818318753/work](file:///\\home\conda\feedstock_root\build_artifacts\argon2-cffi_1692818318753\work)

argon2-cffi-bindings @ [file:///D:/bld/argon2-cffi-bindings_1695386877556/work](file:///D:\bld\argon2-cffi-bindings_1695386877556\work)

arrow @ [file:///home/conda/feedstock_root/build_artifacts/arrow_1696128962909/work](file:///\\home\conda\feedstock_root\build_artifacts\arrow_1696128962909\work)

astor @ [file:///home/conda/feedstock_root/build_artifacts/astor_1593610464257/work](file:///\\home\conda\feedstock_root\build_artifacts\astor_1593610464257\work)

astropy @ [file:///D:/bld/astropy_1711553114901/work](file:///D:\bld\astropy_1711553114901\work)

astropy-iers-data @ [file:///home/conda/feedstock_root/build_artifacts/astropy-iers-data_1725253707459/work](file:///\\home\conda\feedstock_root\build_artifacts\astropy-iers-data_1725253707459\work)

asttokens @ [file:///home/conda/feedstock_root/build_artifacts/asttokens_1698341106958/work](file:///\\home\conda\feedstock_root\build_artifacts\asttokens_1698341106958\work)

astunparse @ [file:///home/conda/feedstock_root/build_artifacts/astunparse_1610696312422/work](file:///\\home\conda\feedstock_root\build_artifacts\astunparse_1610696312422\work)

async-lru @ [file:///home/conda/feedstock_root/build_artifacts/async-lru_1690563019058/work](file:///\\home\conda\feedstock_root\build_artifacts\async-lru_1690563019058\work)

async-timeout @ [file:///home/conda/feedstock_root/build_artifacts/async-timeout_1691763562544/work](file:///\\home\conda\feedstock_root\build_artifacts\async-timeout_1691763562544\work)

attrs @ [file:///home/conda/feedstock_root/build_artifacts/attrs_1722977137225/work](file:///\\home\conda\feedstock_root\build_artifacts\attrs_1722977137225\work)

Babel @ [file:///home/conda/feedstock_root/build_artifacts/babel_1702422572539/work](file:///\\home\conda\feedstock_root\build_artifacts\babel_1702422572539\work)

beautifulsoup4 @ [file:///home/conda/feedstock_root/build_artifacts/beautifulsoup4_1705564648255/work](file:///\\home\conda\feedstock_root\build_artifacts\beautifulsoup4_1705564648255\work)

bleach @ [file:///home/conda/feedstock_root/build_artifacts/bleach_1696630167146/work](file:///\\home\conda\feedstock_root\build_artifacts\bleach_1696630167146\work)

blinker @ [file:///home/conda/feedstock_root/build_artifacts/blinker_1715091184126/work](file:///\\home\conda\feedstock_root\build_artifacts\blinker_1715091184126\work)

blis @ [file:///D:/bld/cython-blis_1696148980119/work](file:///D:\bld\cython-blis_1696148980119\work)

Brotli @ [file:///D:/bld/brotli-split_1687884251065/work](file:///D:\bld\brotli-split_1687884251065\work)

cached-property @ [file:///home/conda/feedstock_root/build_artifacts/cached_property_1615209429212/work](file:///\\home\conda\feedstock_root\build_artifacts\cached_property_1615209429212\work)

cachetools @ [file:///home/conda/feedstock_root/build_artifacts/cachetools_1633010882559/work](file:///\\home\conda\feedstock_root\build_artifacts\cachetools_1633010882559\work)

catalogue @ [file:///D:/bld/catalogue_1695626463227/work](file:///D:\bld\catalogue_1695626463227\work)

certifi @ [file:///home/conda/feedstock_root/build_artifacts/certifi_1725278078093/work/certifi](file:///\\home\conda\feedstock_root\build_artifacts\certifi_1725278078093\work\certifi)

cffi @ [file:///D:/bld/cffi_1723018531537/work](file:///D:\bld\cffi_1723018531537\work)

charset-normalizer @ [file:///home/conda/feedstock_root/build_artifacts/charset-normalizer_1698833585322/work](file:///\\home\conda\feedstock_root\build_artifacts\charset-normalizer_1698833585322\work)

chromedriver-py==127.0.6533.88

click==7.1.2

cloudpathlib @ [file:///home/conda/feedstock_root/build_artifacts/cloudpathlib-meta_1708995864616/work](file:///\\home\conda\feedstock_root\build_artifacts\cloudpathlib-meta_1708995864616\work)

colorama @ [file:///home/conda/feedstock_root/build_artifacts/colorama_1666700638685/work](file:///\\home\conda\feedstock_root\build_artifacts\colorama_1666700638685\work)

comm @ [file:///home/conda/feedstock_root/build_artifacts/comm_1710320294760/work](file:///\\home\conda\feedstock_root\build_artifacts\comm_1710320294760\work)

confection @ [file:///D:/bld/confection_1701179257764/work](file:///D:\bld\confection_1701179257764\work)

contourpy @ [file:///D:/bld/contourpy_1712430020791/work](file:///D:\bld\contourpy_1712430020791\work)

cryptography @ [file:///D:/bld/cryptography-split_1672672551806/work](file:///D:\bld\cryptography-split_1672672551806\work)

cycler @ [file:///home/conda/feedstock_root/build_artifacts/cycler_1696677705766/work](file:///\\home\conda\feedstock_root\build_artifacts\cycler_1696677705766\work)

cymem @ [file:///D:/bld/cymem_1695443662923/work](file:///D:\bld\cymem_1695443662923\work)

dataclasses @ [file:///home/conda/feedstock_root/build_artifacts/dataclasses_1628958434797/work](file:///\\home\conda\feedstock_root\build_artifacts\dataclasses_1628958434797\work)

datasets @ [file:///home/conda/feedstock_root/build_artifacts/datasets_1643849955262/work](file:///\\home\conda\feedstock_root\build_artifacts\datasets_1643849955262\work)

debugpy @ [file:///D:/bld/debugpy_1722923831604/work](file:///D:\bld\debugpy_1722923831604\work)

decorator @ [file:///home/conda/feedstock_root/build_artifacts/decorator_1641555617451/work](file:///\\home\conda\feedstock_root\build_artifacts\decorator_1641555617451\work)

defusedxml @ [file:///home/conda/feedstock_root/build_artifacts/defusedxml_1615232257335/work](file:///\\home\conda\feedstock_root\build_artifacts\defusedxml_1615232257335\work)

dill @ [file:///home/conda/feedstock_root/build_artifacts/dill_1706434688412/work](file:///\\home\conda\feedstock_root\build_artifacts\dill_1706434688412\work)

en-core-web-md @ <https://github.com/explosion/spacy-models/releases/download/en_core_web_md-3.7.1/en_core_web_md-3.7.1-py3-none-any.whl#sha256=6a0f857a2b4d219c6fa17d455f82430b365bf53171a2d919b9376e5dc9be032e>

entrypoints @ [file:///home/conda/feedstock_root/build_artifacts/entrypoints_1643888246732/work](file:///\\home\conda\feedstock_root\build_artifacts\entrypoints_1643888246732\work)

et-xmlfile @ [file:///home/conda/feedstock_root/build_artifacts/et_xmlfile_1674664118162/work](file:///\\home\conda\feedstock_root\build_artifacts\et_xmlfile_1674664118162\work)

exceptiongroup @ [file:///home/conda/feedstock_root/build_artifacts/exceptiongroup_1720869315914/work](file:///\\home\conda\feedstock_root\build_artifacts\exceptiongroup_1720869315914\work)

executing @ [file:///home/conda/feedstock_root/build_artifacts/executing_1698579936712/work](file:///\\home\conda\feedstock_root\build_artifacts\executing_1698579936712\work)

fastjsonschema @ [file:///home/conda/feedstock_root/build_artifacts/python-fastjsonschema_1718477020893/work/dist](file:///\\home\conda\feedstock_root\build_artifacts\python-fastjsonschema_1718477020893\work\dist)

filelock @ [file:///home/conda/feedstock_root/build_artifacts/filelock_1719088281970/work](file:///\\home\conda\feedstock_root\build_artifacts\filelock_1719088281970\work)

flatbuffers @ [file:///home/conda/feedstock_root/build_artifacts/python-flatbuffers_1617723079010/work](file:///\\home\conda\feedstock_root\build_artifacts\python-flatbuffers_1617723079010\work)

fonttools @ [file:///D:/bld/fonttools_1720359129126/work](file:///D:\bld\fonttools_1720359129126\work)

fqdn @ [file:///home/conda/feedstock_root/build_artifacts/fqdn_1638810296540/work/dist](file:///\\home\conda\feedstock_root\build_artifacts\fqdn_1638810296540\work\dist)

frozenlist @ [file:///D:/bld/frozenlist_1702645622997/work](file:///D:\bld\frozenlist_1702645622997\work)

fsspec @ [file:///home/conda/feedstock_root/build_artifacts/fsspec_1719514913127/work](file:///\\home\conda\feedstock_root\build_artifacts\fsspec_1719514913127\work)

gast @ [file:///home/conda/feedstock_root/build_artifacts/gast_1596839682936/work](file:///\\home\conda\feedstock_root\build_artifacts\gast_1596839682936\work)

google-auth @ [file:///home/conda/feedstock_root/build_artifacts/google-auth_1629296548061/work](file:///\\home\conda\feedstock_root\build_artifacts\google-auth_1629296548061\work)

google-auth-oauthlib @ [file:///home/conda/feedstock_root/build_artifacts/google-auth-oauthlib_1630497468950/work](file:///\\home\conda\feedstock_root\build_artifacts\google-auth-oauthlib_1630497468950\work)

google-pasta @ [file:///home/conda/feedstock_root/build_artifacts/google-pasta_1722873999312/work](file:///\\home\conda\feedstock_root\build_artifacts\google-pasta_1722873999312\work)

grpcio @ [file:///D:/bld/grpcio_1653139066827/work](file:///D:\bld\grpcio_1653139066827\work)

h11 @ [file:///home/conda/feedstock_root/build_artifacts/h11_1664132893548/work](file:///\\home\conda\feedstock_root\build_artifacts\h11_1664132893548\work)

h2 @ [file:///home/conda/feedstock_root/build_artifacts/h2_1634280454336/work](file:///\\home\conda\feedstock_root\build_artifacts\h2_1634280454336\work)

h5py @ [file:///D:/bld/h5py_1660488244542/work](file:///D:\bld\h5py_1660488244542\work)

hpack==4.0.0

html5lib==1.1

httpcore @ [file:///home/conda/feedstock_root/build_artifacts/httpcore_1711596990900/work](file:///\\home\conda\feedstock_root\build_artifacts\httpcore_1711596990900\work)

httpx @ [file:///home/conda/feedstock_root/build_artifacts/httpx_1708530890843/work](file:///\\home\conda\feedstock_root\build_artifacts\httpx_1708530890843\work)

huggingface_hub @ [file:///home/conda/feedstock_root/build_artifacts/huggingface_hub_1722613646665/work](file:///\\home\conda\feedstock_root\build_artifacts\huggingface_hub_1722613646665\work)

hyperframe @ [file:///home/conda/feedstock_root/build_artifacts/hyperframe_1619110129307/work](file:///\\home\conda\feedstock_root\build_artifacts\hyperframe_1619110129307\work)

idna @ [file:///home/conda/feedstock_root/build_artifacts/idna_1713279365350/work](file:///\\home\conda\feedstock_root\build_artifacts\idna_1713279365350\work)

imagecodecs @ [file:///D:/bld/imagecodecs_1668867797884/work](file:///D:\bld\imagecodecs_1668867797884\work)

imageio @ [file:///home/conda/feedstock_root/build_artifacts/imageio_1724069053555/work](file:///\\home\conda\feedstock_root\build_artifacts\imageio_1724069053555\work)

importlib_metadata @ [file:///home/conda/feedstock_root/build_artifacts/importlib-metadata_1721856510709/work](file:///\\home\conda\feedstock_root\build_artifacts\importlib-metadata_1721856510709\work)

importlib_resources @ [file:///home/conda/feedstock_root/build_artifacts/importlib_resources_1723702735258/work](file:///\\home\conda\feedstock_root\build_artifacts\importlib_resources_1723702735258\work)

imutils==0.5.4

ipykernel @ [file:///D:/bld/ipykernel_1719845595208/work](file:///D:\bld\ipykernel_1719845595208\work)

ipython @ [file:///D:/bld/ipython_1701831845989/work](file:///D:\bld\ipython_1701831845989\work)

ipython-genutils==0.2.0

ipywidgets @ [file:///home/conda/feedstock_root/build_artifacts/ipywidgets_1716897651763/work](file:///\\home\conda\feedstock_root\build_artifacts\ipywidgets_1716897651763\work)

isoduration @ [file:///home/conda/feedstock_root/build_artifacts/isoduration_1638811571363/work/dist](file:///\\home\conda\feedstock_root\build_artifacts\isoduration_1638811571363\work\dist)

jedi @ [file:///home/conda/feedstock_root/build_artifacts/jedi_1696326070614/work](file:///\\home\conda\feedstock_root\build_artifacts\jedi_1696326070614\work)

Jinja2 @ [file:///home/conda/feedstock_root/build_artifacts/jinja2_1715127149914/work](file:///\\home\conda\feedstock_root\build_artifacts\jinja2_1715127149914\work)

joblib @ [file:///home/conda/feedstock_root/build_artifacts/joblib_1714665484399/work](file:///\\home\conda\feedstock_root\build_artifacts\joblib_1714665484399\work)

json5 @ [file:///home/conda/feedstock_root/build_artifacts/json5_1712986206667/work](file:///\\home\conda\feedstock_root\build_artifacts\json5_1712986206667\work)

jsonpointer @ [file:///D:/bld/jsonpointer_1718283513780/work](file:///D:\bld\jsonpointer_1718283513780\work)

jsonschema @ [file:///home/conda/feedstock_root/build_artifacts/jsonschema_1720529478715/work](file:///\\home\conda\feedstock_root\build_artifacts\jsonschema_1720529478715\work)

jsonschema-specifications @ [file:///tmp/tmpkv1z7p57/src](file:///\\tmp\tmpkv1z7p57\src)

jupyter-events @ [file:///home/conda/feedstock_root/build_artifacts/jupyter_events_1710805637316/work](file:///\\home\conda\feedstock_root\build_artifacts\jupyter_events_1710805637316\work)

jupyter-highlight-selected-word==0.2.0

jupyter-lsp @ [file:///home/conda/feedstock_root/build_artifacts/jupyter-lsp-meta_1712707420468/work/jupyter-lsp](file:///\\home\conda\feedstock_root\build_artifacts\jupyter-lsp-meta_1712707420468\work\jupyter-lsp)

jupyter_client @ [file:///home/conda/feedstock_root/build_artifacts/jupyter_client_1716472197302/work](file:///\\home\conda\feedstock_root\build_artifacts\jupyter_client_1716472197302\work)

jupyter_contrib_core==0.4.2

jupyter_core @ [file:///D:/bld/jupyter_core_1710257377578/work](file:///D:\bld\jupyter_core_1710257377578\work)

jupyter_nbextensions_configurator==0.6.4

jupyter_server @ [file:///home/conda/feedstock_root/build_artifacts/jupyter_server_1720816649297/work](file:///\\home\conda\feedstock_root\build_artifacts\jupyter_server_1720816649297\work)

jupyter_server_terminals @ [file:///home/conda/feedstock_root/build_artifacts/jupyter_server_terminals_1710262634903/work](file:///\\home\conda\feedstock_root\build_artifacts\jupyter_server_terminals_1710262634903\work)

jupyterlab @ [file:///home/conda/feedstock_root/build_artifacts/jupyterlab_1721396525904/work](file:///\\home\conda\feedstock_root\build_artifacts\jupyterlab_1721396525904\work)

jupyterlab_pygments @ [file:///home/conda/feedstock_root/build_artifacts/jupyterlab_pygments_1707149102966/work](file:///\\home\conda\feedstock_root\build_artifacts\jupyterlab_pygments_1707149102966\work)

jupyterlab_server @ [file:///home/conda/feedstock_root/build_artifacts/jupyterlab_server-split_1721163288448/work](file:///\\home\conda\feedstock_root\build_artifacts\jupyterlab_server-split_1721163288448\work)

jupyterlab_widgets @ [file:///home/conda/feedstock_root/build_artifacts/jupyterlab_widgets_1716891641122/work](file:///\\home\conda\feedstock_root\build_artifacts\jupyterlab_widgets_1716891641122\work)

keras @ [file:///home/conda/feedstock_root/build_artifacts/keras_1637159014053/work/keras-2.6.0-py2.py3-none-any.whl](file:///\\home\conda\feedstock_root\build_artifacts\keras_1637159014053\work\keras-2.6.0-py2.py3-none-any.whl)

Keras-Preprocessing @ [file:///home/conda/feedstock_root/build_artifacts/keras-preprocessing_1610713559828/work](file:///\\home\conda\feedstock_root\build_artifacts\keras-preprocessing_1610713559828\work)

kiwisolver @ [file:///D:/bld/kiwisolver_1695380110085/work](file:///D:\bld\kiwisolver_1695380110085\work)

langcodes @ [file:///home/conda/feedstock_root/build_artifacts/langcodes_1714235526219/work](file:///\\home\conda\feedstock_root\build_artifacts\langcodes_1714235526219\work)

language_data @ [file:///home/conda/feedstock_root/build_artifacts/language-data_1714193818885/work](file:///\\home\conda\feedstock_root\build_artifacts\language-data_1714193818885\work)

lazy_loader @ [file:///home/conda/feedstock_root/build_artifacts/lazy-loader_1723774329602/work](file:///\\home\conda\feedstock_root\build_artifacts\lazy-loader_1723774329602\work)

lxml==5.2.2

marisa-trie @ [file:///D:/bld/marisa-trie_1706566698330/work](file:///D:\bld\marisa-trie_1706566698330\work)

Markdown @ [file:///home/conda/feedstock_root/build_artifacts/markdown_1710435156458/work](file:///\\home\conda\feedstock_root\build_artifacts\markdown_1710435156458\work)

markdown-it-py @ [file:///home/conda/feedstock_root/build_artifacts/markdown-it-py_1686175045316/work](file:///\\home\conda\feedstock_root\build_artifacts\markdown-it-py_1686175045316\work)

MarkupSafe @ [file:///D:/bld/markupsafe_1706900063757/work](file:///D:\bld\markupsafe_1706900063757\work)

matplotlib==3.9.1

matplotlib-inline @ [file:///home/conda/feedstock_root/build_artifacts/matplotlib-inline_1713250518406/work](file:///\\home\conda\feedstock_root\build_artifacts\matplotlib-inline_1713250518406\work)

mdurl @ [file:///home/conda/feedstock_root/build_artifacts/mdurl_1704317613764/work](file:///\\home\conda\feedstock_root\build_artifacts\mdurl_1704317613764\work)

mistune @ [file:///home/conda/feedstock_root/build_artifacts/mistune_1698947099619/work](file:///\\home\conda\feedstock_root\build_artifacts\mistune_1698947099619\work)

morphsnakes==2.0.1

mpmath @ [file:///home/conda/feedstock_root/build_artifacts/mpmath_1678228039184/work](file:///\\home\conda\feedstock_root\build_artifacts\mpmath_1678228039184\work)

multidict @ [file:///D:/bld/multidict_1707040875863/work](file:///D:\bld\multidict_1707040875863\work)

multiprocess @ [file:///D:/bld/multiprocess_1706514757841/work](file:///D:\bld\multiprocess_1706514757841\work)

munkres==1.1.4

murmurhash @ [file:///D:/bld/murmurhash_1695449904203/work](file:///D:\bld\murmurhash_1695449904203\work)

natsort==8.4.0

nbclient @ [file:///home/conda/feedstock_root/build_artifacts/nbclient_1710317608672/work](file:///\\home\conda\feedstock_root\build_artifacts\nbclient_1710317608672\work)

nbconvert @ [file:///home/conda/feedstock_root/build_artifacts/nbconvert-meta_1718135430380/work](file:///\\home\conda\feedstock_root\build_artifacts\nbconvert-meta_1718135430380\work)

nbformat @ [file:///home/conda/feedstock_root/build_artifacts/nbformat_1712238998817/work](file:///\\home\conda\feedstock_root\build_artifacts\nbformat_1712238998817\work)

nest_asyncio @ [file:///home/conda/feedstock_root/build_artifacts/nest-asyncio_1705850609492/work](file:///\\home\conda\feedstock_root\build_artifacts\nest-asyncio_1705850609492\work)

networkx @ [file:///home/conda/feedstock_root/build_artifacts/networkx_1697694336581/work](file:///\\home\conda\feedstock_root\build_artifacts\networkx_1697694336581\work)

nltk @ [file:///home/conda/feedstock_root/build_artifacts/nltk_1724022741962/work](file:///\\home\conda\feedstock_root\build_artifacts\nltk_1724022741962\work)

notebook @ [file:///home/conda/feedstock_root/build_artifacts/notebook_1717767745914/work](file:///\\home\conda\feedstock_root\build_artifacts\notebook_1717767745914\work)

notebook_shim @ [file:///home/conda/feedstock_root/build_artifacts/notebook-shim_1707957777232/work](file:///\\home\conda\feedstock_root\build_artifacts\notebook-shim_1707957777232\work)

numpy @ [file:///D:/bld/numpy_1666788367701/work](file:///D:\bld\numpy_1666788367701\work)

oauthlib @ [file:///home/conda/feedstock_root/build_artifacts/oauthlib_1666056362788/work](file:///\\home\conda\feedstock_root\build_artifacts\oauthlib_1666056362788\work)

openpyxl @ [file:///D:/bld/openpyxl_1723459205255/work](file:///D:\bld\openpyxl_1723459205255\work)

opt-einsum @ [file:///home/conda/feedstock_root/build_artifacts/opt_einsum_1696448916724/work](file:///\\home\conda\feedstock_root\build_artifacts\opt_einsum_1696448916724\work)

outcome @ [file:///home/conda/feedstock_root/build_artifacts/outcome_1698324044871/work](file:///\\home\conda\feedstock_root\build_artifacts\outcome_1698324044871\work)

overrides @ [file:///home/conda/feedstock_root/build_artifacts/overrides_1706394519472/work](file:///\\home\conda\feedstock_root\build_artifacts\overrides_1706394519472\work)

packaging @ [file:///home/conda/feedstock_root/build_artifacts/packaging_1718189413536/work](file:///\\home\conda\feedstock_root\build_artifacts\packaging_1718189413536\work)

pandas @ [file:///D:/bld/pandas_1715897793875/work](file:///D:\bld\pandas_1715897793875\work)

pandocfilters @ [file:///home/conda/feedstock_root/build_artifacts/pandocfilters_1631603243851/work](file:///\\home\conda\feedstock_root\build_artifacts\pandocfilters_1631603243851\work)

parso @ [file:///home/conda/feedstock_root/build_artifacts/parso_1712320355065/work](file:///\\home\conda\feedstock_root\build_artifacts\parso_1712320355065\work)

pathy @ [file:///C:/b/abs_16nuqmi0a0/croot/pathy_1703688148677/work](file:///C:\b\abs_16nuqmi0a0\croot\pathy_1703688148677\work)

patsy @ [file:///home/conda/feedstock_root/build_artifacts/patsy_1704469236901/work](file:///\\home\conda\feedstock_root\build_artifacts\patsy_1704469236901\work)

pdf2image @ [file:///home/conda/feedstock_root/build_artifacts/pdf2image_1704665640257/work](file:///\\home\conda\feedstock_root\build_artifacts\pdf2image_1704665640257\work)

pdfkit==1.0.0

pickleshare @ [file:///home/conda/feedstock_root/build_artifacts/pickleshare_1602536217715/work](file:///\\home\conda\feedstock_root\build_artifacts\pickleshare_1602536217715\work)

Pillow @ [file:///C:/b/abs_153xikw91n/croot/pillow_1695134603563/work](file:///C:\b\abs_153xikw91n\croot\pillow_1695134603563\work)

pkgutil_resolve_name @ [file:///home/conda/feedstock_root/build_artifacts/pkgutil-resolve-name_1694617248815/work](file:///\\home\conda\feedstock_root\build_artifacts\pkgutil-resolve-name_1694617248815\work)

platformdirs @ [file:///home/conda/feedstock_root/build_artifacts/platformdirs_1715777629804/work](file:///\\home\conda\feedstock_root\build_artifacts\platformdirs_1715777629804\work)

preshed @ [file:///D:/bld/preshed_1695645036794/work](file:///D:\bld\preshed_1695645036794\work)

prometheus_client @ [file:///home/conda/feedstock_root/build_artifacts/prometheus_client_1707932675456/work](file:///\\home\conda\feedstock_root\build_artifacts\prometheus_client_1707932675456\work)

prompt_toolkit @ [file:///home/conda/feedstock_root/build_artifacts/prompt-toolkit_1718047967974/work](file:///\\home\conda\feedstock_root\build_artifacts\prompt-toolkit_1718047967974\work)

protobuf==3.17.2

psutil @ [file:///D:/bld/psutil_1719274706746/work](file:///D:\bld\psutil_1719274706746\work)

pure_eval @ [file:///home/conda/feedstock_root/build_artifacts/pure_eval_1721585709575/work](file:///\\home\conda\feedstock_root\build_artifacts\pure_eval_1721585709575\work)

pyarrow==3.0.0

pyasn1 @ [file:///home/conda/feedstock_root/build_artifacts/pyasn1_1713209357222/work](file:///\\home\conda\feedstock_root\build_artifacts\pyasn1_1713209357222\work)

pyasn1_modules @ [file:///home/conda/feedstock_root/build_artifacts/pyasn1-modules_1713209683338/work](file:///\\home\conda\feedstock_root\build_artifacts\pyasn1-modules_1713209683338\work)

pycparser @ [file:///home/conda/feedstock_root/build_artifacts/pycparser_1711811537435/work](file:///\\home\conda\feedstock_root\build_artifacts\pycparser_1711811537435\work)

pydantic==1.10.17

pydantic_core @ [file:///D:/bld/pydantic-core_1720041357218/work](file:///D:\bld\pydantic-core_1720041357218\work)

pydicom==2.3.0

pyerfa @ [file:///D:/bld/pyerfa_1715855025873/work](file:///D:\bld\pyerfa_1715855025873\work)

Pygments @ [file:///home/conda/feedstock_root/build_artifacts/pygments_1714846767233/work](file:///\\home\conda\feedstock_root\build_artifacts\pygments_1714846767233\work)

PyJWT @ [file:///home/conda/feedstock_root/build_artifacts/pyjwt_1722701264352/work](file:///\\home\conda\feedstock_root\build_artifacts\pyjwt_1722701264352\work)

pyOpenSSL @ [file:///home/conda/feedstock_root/build_artifacts/pyopenssl_1685514481738/work](file:///\\home\conda\feedstock_root\build_artifacts\pyopenssl_1685514481738\work)

pyparsing @ [file:///home/conda/feedstock_root/build_artifacts/pyparsing_1709721012883/work](file:///\\home\conda\feedstock_root\build_artifacts\pyparsing_1709721012883\work)

PyPDF2 @ [file:///home/conda/feedstock_root/build_artifacts/pypdf2_1723906867750/work](file:///\\home\conda\feedstock_root\build_artifacts\pypdf2_1723906867750\work)

pyperclip==1.9.0

PyQt5==5.12.3

PyQt5_sip==4.19.18

PyQtChart==5.12

PyQtWebEngine==5.12.1

PySocks @ [file:///D:/bld/pysocks_1661604991356/work](file:///D:\bld\pysocks_1661604991356\work)

pytesseract @ [file:///home/conda/feedstock_root/build_artifacts/pytesseract_1697399219056/work](file:///\\home\conda\feedstock_root\build_artifacts\pytesseract_1697399219056\work)

python-dateutil @ [file:///home/conda/feedstock_root/build_artifacts/python-dateutil_1709299778482/work](file:///\\home\conda\feedstock_root\build_artifacts\python-dateutil_1709299778482\work)

python-docx @ [file:///home/conda/feedstock_root/build_artifacts/python-docx_1714608302440/work](file:///\\home\conda\feedstock_root\build_artifacts\python-docx_1714608302440\work)

python-json-logger @ [file:///home/conda/feedstock_root/build_artifacts/python-json-logger_1677079630776/work](file:///\\home\conda\feedstock_root\build_artifacts\python-json-logger_1677079630776\work)

python-pptx==1.0.2

pytz @ [file:///home/conda/feedstock_root/build_artifacts/pytz_1706886791323/work](file:///\\home\conda\feedstock_root\build_artifacts\pytz_1706886791323\work)

pyu2f @ [file:///home/conda/feedstock_root/build_artifacts/pyu2f_1604248910016/work](file:///\\home\conda\feedstock_root\build_artifacts\pyu2f_1604248910016\work)

PyWavelets==1.6.0

pywin32==306

pywinpty @ [file:///D:/bld/pywinpty_1708993010363/work/target/wheels/pywinpty-2.0.13-cp39-none-win_amd64.whl#sha256=84b4ae7fb10140f362ee7ce26d822343cddcefa8007d779a0392e447cd05b281](file:///D:\bld\pywinpty_1708993010363\work\target\wheels\pywinpty-2.0.13-cp39-none-win_amd64.whl#sha256=84b4ae7fb10140f362ee7ce26d822343cddcefa8007d779a0392e447cd05b281)

PyYAML @ [file:///D:/bld/pyyaml_1723018304772/work](file:///D:\bld\pyyaml_1723018304772\work)

pyzmq @ [file:///D:/bld/pyzmq_1722971783154/work](file:///D:\bld\pyzmq_1722971783154\work)

referencing @ [file:///home/conda/feedstock_root/build_artifacts/referencing_1714619483868/work](file:///\\home\conda\feedstock_root\build_artifacts\referencing_1714619483868\work)

regex @ [file:///D:/bld/regex_1721873087396/work](file:///D:\bld\regex_1721873087396\work)

requests @ [file:///home/conda/feedstock_root/build_artifacts/requests_1717057054362/work](file:///\\home\conda\feedstock_root\build_artifacts\requests_1717057054362\work)

requests-oauthlib @ [file:///home/conda/feedstock_root/build_artifacts/requests-oauthlib_1711290127547/work](file:///\\home\conda\feedstock_root\build_artifacts\requests-oauthlib_1711290127547\work)

rfc3339-validator @ [file:///home/conda/feedstock_root/build_artifacts/rfc3339-validator_1638811747357/work](file:///\\home\conda\feedstock_root\build_artifacts\rfc3339-validator_1638811747357\work)

rfc3986-validator @ [file:///home/conda/feedstock_root/build_artifacts/rfc3986-validator_1598024191506/work](file:///\\home\conda\feedstock_root\build_artifacts\rfc3986-validator_1598024191506\work)

rich @ [file:///home/conda/feedstock_root/build_artifacts/rich-split_1709150387247/work/dist](file:///\\home\conda\feedstock_root\build_artifacts\rich-split_1709150387247\work\dist)

rpds-py @ [file:///D:/bld/rpds-py_1723039243945/work](file:///D:\bld\rpds-py_1723039243945\work)

rsa @ [file:///home/conda/feedstock_root/build_artifacts/rsa_1658328885051/work](file:///\\home\conda\feedstock_root\build_artifacts\rsa_1658328885051\work)

safetensors @ [file:///D:/bld/safetensors_1722938011551/work](file:///D:\bld\safetensors_1722938011551\work)

scikit-image @ [file:///D:/bld/scikit-image_1723842294643/work](file:///D:\bld\scikit-image_1723842294643\work)

scikit-learn @ [file:///D:/bld/scikit-learn_1719998210371/work/dist/scikit_learn-1.5.1-cp39-cp39-win_amd64.whl#sha256=7580274376572e082b04fda4325d8ef71ffaf8e5854db686a13b5d252286ac91](file:///D:\bld\scikit-learn_1719998210371\work\dist\scikit_learn-1.5.1-cp39-cp39-win_amd64.whl#sha256=7580274376572e082b04fda4325d8ef71ffaf8e5854db686a13b5d252286ac91)

scipy==1.9.1

selenium @ [file:///home/conda/feedstock_root/build_artifacts/selenium_1726926491457/work](file:///\\home\conda\feedstock_root\build_artifacts\selenium_1726926491457\work)

Send2Trash @ [file:///D:/bld/send2trash_1712585174948/work](file:///D:\bld\send2trash_1712585174948\work)

sentence-transformers==3.0.1

shellingham @ [file:///home/conda/feedstock_root/build_artifacts/shellingham_1698144360966/work](file:///\\home\conda\feedstock_root\build_artifacts\shellingham_1698144360966\work)

SimpleITK==2.2.1

six @ [file:///home/conda/feedstock_root/build_artifacts/six_1620240208055/work](file:///\\home\conda\feedstock_root\build_artifacts\six_1620240208055\work)

smart-open @ [file:///home/conda/feedstock_root/build_artifacts/smart_open_split_1694066705667/work/dist](file:///\\home\conda\feedstock_root\build_artifacts\smart_open_split_1694066705667\work\dist)

sniffio @ [file:///home/conda/feedstock_root/build_artifacts/sniffio_1708952932303/work](file:///\\home\conda\feedstock_root\build_artifacts\sniffio_1708952932303\work)

sortedcontainers @ [file:///home/conda/feedstock_root/build_artifacts/sortedcontainers_1621217038088/work](file:///\\home\conda\feedstock_root\build_artifacts\sortedcontainers_1621217038088\work)

soupsieve @ [file:///home/conda/feedstock_root/build_artifacts/soupsieve_1693929250441/work](file:///\\home\conda\feedstock_root\build_artifacts\soupsieve_1693929250441\work)

spacy==3.7.5

spacy-legacy @ [file:///home/conda/feedstock_root/build_artifacts/spacy-legacy_1674550301837/work](file:///\\home\conda\feedstock_root\build_artifacts\spacy-legacy_1674550301837\work)

spacy-loggers @ [file:///home/conda/feedstock_root/build_artifacts/spacy-loggers_1694527114282/work](file:///\\home\conda\feedstock_root\build_artifacts\spacy-loggers_1694527114282\work)

srsly @ [file:///D:/bld/srsly_1695654290456/work](file:///D:\bld\srsly_1695654290456\work)

stack-data @ [file:///home/conda/feedstock_root/build_artifacts/stack_data_1669632077133/work](file:///\\home\conda\feedstock_root\build_artifacts\stack_data_1669632077133\work)

statsmodels @ [file:///D:/bld/statsmodels_1715941390207/work](file:///D:\bld\statsmodels_1715941390207\work)

sympy @ [file:///home/conda/feedstock_root/build_artifacts/sympy_1723500263401/work](file:///\\home\conda\feedstock_root\build_artifacts\sympy_1723500263401\work)

tabulate @ [file:///home/conda/feedstock_root/build_artifacts/tabulate_1665138452165/work](file:///\\home\conda\feedstock_root\build_artifacts\tabulate_1665138452165\work)

tensorboard @ [file:///home/conda/feedstock_root/build_artifacts/tensorboard_1629677129676/work/tensorboard-2.6.0-py3-none-any.whl](file:///\\home\conda\feedstock_root\build_artifacts\tensorboard_1629677129676\work\tensorboard-2.6.0-py3-none-any.whl)

tensorboard-data-server @ [file:///D:/bld/tensorboard-data-server_1670043950676/work/tensorboard_data_server-0.6.1-py3-none-any.whl](file:///D:\bld\tensorboard-data-server_1670043950676\work\tensorboard_data_server-0.6.1-py3-none-any.whl)

tensorboard-plugin-wit @ [file:///home/conda/feedstock_root/build_artifacts/tensorboard-plugin-wit_1641458951060/work/tensorboard_plugin_wit-1.8.1-py3-none-any.whl](file:///\\home\conda\feedstock_root\build_artifacts\tensorboard-plugin-wit_1641458951060\work\tensorboard_plugin_wit-1.8.1-py3-none-any.whl)

tensorflow==2.6.0

tensorflow-estimator @ [file:///home/builder/adipietro/tf/tensorflow-estimator_1630508970172/work/tensorflow_estimator-2.6.0-py2.py3-none-any.whl](file:///\\home\builder\adipietro\tf\tensorflow-estimator_1630508970172\work\tensorflow_estimator-2.6.0-py2.py3-none-any.whl)

termcolor @ [file:///home/conda/feedstock_root/build_artifacts/termcolor_1704357939450/work](file:///\\home\conda\feedstock_root\build_artifacts\termcolor_1704357939450\work)

terminado @ [file:///D:/bld/terminado_1710262761616/work](file:///D:\bld\terminado_1710262761616\work)

thinc==8.2.5

threadpoolctl @ [file:///home/conda/feedstock_root/build_artifacts/threadpoolctl_1714400101435/work](file:///\\home\conda\feedstock_root\build_artifacts\threadpoolctl_1714400101435\work)

tifffile @ [file:///home/conda/feedstock_root/build_artifacts/tifffile_1665588749940/work](file:///\\home\conda\feedstock_root\build_artifacts\tifffile_1665588749940\work)

tinycss2 @ [file:///home/conda/feedstock_root/build_artifacts/tinycss2_1713974937325/work](file:///\\home\conda\feedstock_root\build_artifacts\tinycss2_1713974937325\work)

tokenizers @ [file:///D:/bld/tokenizers_1713402858760/work/bindings/python](file:///D:\bld\tokenizers_1713402858760\work\bindings\python)

tomli @ [file:///home/conda/feedstock_root/build_artifacts/tomli_1644342247877/work](file:///\\home\conda\feedstock_root\build_artifacts\tomli_1644342247877\work)

torch==2.4.0

torchaudio==2.4.0

tornado @ [file:///D:/bld/tornado_1717722891796/work](file:///D:\bld\tornado_1717722891796\work)

tqdm @ [file:///home/conda/feedstock_root/build_artifacts/tqdm_1722737464726/work](file:///\\home\conda\feedstock_root\build_artifacts\tqdm_1722737464726\work)

traitlets @ [file:///home/conda/feedstock_root/build_artifacts/traitlets_1713535121073/work](file:///\\home\conda\feedstock_root\build_artifacts\traitlets_1713535121073\work)

transformers @ [file:///home/conda/feedstock_root/build_artifacts/transformers_1723002526807/work](file:///\\home\conda\feedstock_root\build_artifacts\transformers_1723002526807\work)

trio @ [file:///D:/bld/trio_1725302466905/work](file:///D:\bld\trio_1725302466905\work)

trio-websocket @ [file:///home/conda/feedstock_root/build_artifacts/trio-websocket_1695816857197/work/dist](file:///\\home\conda\feedstock_root\build_artifacts\trio-websocket_1695816857197\work\dist)

typer @ [file:///home/conda/feedstock_root/build_artifacts/typer_1609874382867/work](file:///\\home\conda\feedstock_root\build_artifacts\typer_1609874382867\work)

types-python-dateutil @ [file:///home/conda/feedstock_root/build_artifacts/types-python-dateutil_1710589910274/work](file:///\\home\conda\feedstock_root\build_artifacts\types-python-dateutil_1710589910274\work)

typing-utils @ [file:///home/conda/feedstock_root/build_artifacts/typing_utils_1622899189314/work](file:///\\home\conda\feedstock_root\build_artifacts\typing_utils_1622899189314\work)

typing_extensions @ [file:///home/conda/feedstock_root/build_artifacts/typing_extensions_1717802530399/work](file:///\\home\conda\feedstock_root\build_artifacts\typing_extensions_1717802530399\work)

tzdata @ [file:///home/conda/feedstock_root/build_artifacts/python-tzdata_1707747584337/work](file:///\\home\conda\feedstock_root\build_artifacts\python-tzdata_1707747584337\work)

unicodedata2 @ [file:///D:/bld/unicodedata2_1695847967701/work](file:///D:\bld\unicodedata2_1695847967701\work)

uri-template @ [file:///home/conda/feedstock_root/build_artifacts/uri-template_1688655812972/work/dist](file:///\\home\conda\feedstock_root\build_artifacts\uri-template_1688655812972\work\dist)

urllib3 @ [file:///home/conda/feedstock_root/build_artifacts/urllib3_1719391292974/work](file:///\\home\conda\feedstock_root\build_artifacts\urllib3_1719391292974\work)

vtk==9.3.1

wasabi @ [file:///home/conda/feedstock_root/build_artifacts/wasabi_1668249950899/work](file:///\\home\conda\feedstock_root\build_artifacts\wasabi_1668249950899\work)

wcwidth @ [file:///home/conda/feedstock_root/build_artifacts/wcwidth_1704731205417/work](file:///\\home\conda\feedstock_root\build_artifacts\wcwidth_1704731205417\work)

weasel @ [file:///home/conda/feedstock_root/build_artifacts/weasel_1719987001521/work](file:///\\home\conda\feedstock_root\build_artifacts\weasel_1719987001521\work)

webcolors @ [file:///home/conda/feedstock_root/build_artifacts/webcolors_1723294704277/work](file:///\\home\conda\feedstock_root\build_artifacts\webcolors_1723294704277\work)

webencodings @ [file:///home/conda/feedstock_root/build_artifacts/webencodings_1694681268211/work](file:///\\home\conda\feedstock_root\build_artifacts\webencodings_1694681268211\work)

websocket-client @ [file:///home/conda/feedstock_root/build_artifacts/websocket-client_1713923384721/work](file:///\\home\conda\feedstock_root\build_artifacts\websocket-client_1713923384721\work)

Werkzeug @ [file:///home/conda/feedstock_root/build_artifacts/werkzeug_1715000201436/work](file:///\\home\conda\feedstock_root\build_artifacts\werkzeug_1715000201436\work)

widgetsnbextension @ [file:///home/conda/feedstock_root/build_artifacts/widgetsnbextension_1716891659446/work](file:///\\home\conda\feedstock_root\build_artifacts\widgetsnbextension_1716891659446\work)

win-inet-pton @ [file:///D:/bld/win_inet_pton_1667051142467/work](file:///D:\bld\win_inet_pton_1667051142467\work)

wrapt @ [file:///D:/bld/wrapt_1699532935905/work](file:///D:\bld\wrapt_1699532935905\work)

wsproto @ [file:///home/conda/feedstock_root/build_artifacts/wsproto_1661356345548/work](file:///\\home\conda\feedstock_root\build_artifacts\wsproto_1661356345548\work)

XlsxWriter==3.2.0

xxhash @ [file:///D:/bld/python-xxhash_1696486422349/work](file:///D:\bld\python-xxhash_1696486422349\work)

yarl @ [file:///D:/bld/yarl_1705508472464/work](file:///D:\bld\yarl_1705508472464\work)

zipp @ [file:///home/conda/feedstock_root/build_artifacts/zipp_1723591248676/work](file:///\\home\conda\feedstock_root\build_artifacts\zipp_1723591248676\work)

zstandard==0.23.0
